# Lysosomal Cholesterol Accumulation Sensitizes To Acetaminophen Hepatotoxicity by Impairing Mitophagy

**DOI:** 10.1038/srep18017

**Published:** 2015-12-11

**Authors:** Anna Baulies, Vicent Ribas, Susana Núñez, Sandra Torres, Cristina Alarcón-Vila, Laura Martínez, Jo Suda, Maria D. Ybanez, Neil Kaplowitz, Carmen García-Ruiz, Jose C. Fernández-Checa

**Affiliations:** 1 Department of Cell Death and Proliferation, Instituto Investigaciones Biomedicas de Barcelona, CSIC, Barcelona, and Liver Unit-Hospital Clinic-IDIBAPS; 2 Centro de Investigación Biomédica en Red (CIBERehd), Barcelona, Spain; 3 Division of Gastrointestinal and Liver Diseases, Keck School of Medicine, University of Southern California Research Center for Liver Diseases, University of Southern California, Los Angeles, CA 90089-9121, USA; 4 University of Southern California Research Center for Alcohol Liver and Pancreatic Diseases and Cirrhosis, Keck School of Medicine, USC, Los Angeles, CA, USA

## Abstract

The role of lysosomes in acetaminophen (APAP) hepatotoxicity is poorly understood. Here, we investigated the impact of genetic and drug-induced lysosomal cholesterol (LC) accumulation in APAP hepatotoxicity. Acid sphingomyelinase (ASMase)^−/−^ mice exhibit LC accumulation and higher mortality after APAP overdose compared to ASMase^+/+^ littermates. ASMase^−/−^ hepatocytes display lower threshold for APAP-induced cell death and defective fusion of mitochondria-containing autophagosomes with lysosomes, which decreased mitochondrial quality control. LC accumulation in ASMase^+/+^ hepatocytes caused by U18666A reproduces the susceptibility of ASMase^−/−^ hepatocytes to APAP and the impairment in the formation of mitochondria-containing autolysosomes. LC extraction by 25-hydroxycholesterol increased APAP-mediated mitophagy and protected ASMase^−/−^ mice and hepatocytes against APAP hepatotoxicity, effects that were reversed by chloroquine to disrupt autophagy. The regulation of LC by U18666A or 25-hydroxycholesterol did not affect total cellular sphingomyelin content or its lysosomal distribution. Of relevance, amitriptyline-induced ASMase inhibition in human hepatocytes caused LC accumulation, impaired mitophagy and increased susceptibility to APAP. Similar results were observed upon glucocerebrosidase inhibition by conduritol β-epoxide, a cellular model of Gaucher disease. These findings indicate that LC accumulation determines susceptibility to APAP hepatotoxicity by modulating mitophagy, and imply that genetic or drug-mediated ASMase disruption sensitizes to APAP-induced liver injury.

Acetaminophen (APAP), a widely used pain reliever, is a dose-dependent hepatotoxin and a major cause of acute liver failure^1^,^2^. The metabolism of APAP to the reactive metabolite N-acetyl-p-benzo-quinoneimine (NAPQI) triggers hepatocellular death following binding of NAPQI to mitochondrial protein thiols, mitochondrial JNK translocation, induction of mitochondrial permeability pore transition (MPT), generation of reactive oxygen species (ROS) and ATP depletion^3^,^4^,^5^,^6^,^7^,^8^. These events are prevented if enough GSH is available to detoxify NAPQI. Given the crucial role of mitochondria in APAP-induced hepatotoxicity, selective removal of damaged mitochondria is critical to limit liver injury.

Macroautophagy (also referred as autophagy) is a catabolic process that degrades cellular proteins and damaged organelles through the fusion of autophagosomes with lysosomes for the degradation of cargo contents, including damaged mitochondria in a process called mitophagy^9^,^10^,^11^. Recent findings indicated that APAP overdose induces autophagy in both primary cultured mouse hepatocytes and in the mouse liver. Pharmacological induction of autophagy by rapamycin protects against APAP-induced hepatotoxicity^12^,^13^, and the proposed mechanism of protection was the removal of APAP-induced damaged mitochondria.

Lysosomes constitute the primary degradative compartment of the cell that maintain cellular homeostasis and physiological processes^14^. However, very little is known about the role of lysosomes in APAP hepatotoxicity. Lysosomes have been involved in APAP-induced liver injury by a mechanism inducing MPT via lysosomal iron mobilization^15^. Moreover, APAP causes lysosomal instability and cathepsin B release; however, this pathway does not seem to play a significant role in APAP hepatotoxicity^16^.

Acid sphingomyelinase (ASMase) plays an important role in lysosomal membrane turnover through the hydrolysis of sphingomyelin, which results in ceramide generation. ASMase deficiency causes the lysosomal storage disease (LSD) Niemann-Pick type A (NPA), which is characterized primarily by sphingomyelin accumulation and a secondary increase in cholesterol in lysosomes of affected organs (e.g. brain, spleen and liver)^17^. An emerging role of ASMase in autophagy has been recently described^18^. Cells deficient in ASMase exhibit impaired autophagic flux, increased LC3BII levels and enhanced p62 content^19^,^20^. Moreover, ASMase deficiency prevents lysosomal membrane permeabilization caused by palmitic acid and protects against steatohepatitis^19^,^21^.

Here we show that lysosomal cholesterol (LC) accumulation by ASMase deficiency or glucocerebrosidase inhibition by conduritol β-epoxide (CBE), a chemical trigger of Gaucher disease (GD)^22^, exacerbates APAP hepatotoxicity by impairing mitophagy. Importantly, human hepatocytes treated with amitriptyline, a tricyclic antidepressant that inhibits ASMase^23^, exhibit LC accumulation, impaired mitophagy and sensitization to APAP-induced cell death. These findings imply that patients with genetic or pharmacological compromised ASMase activity may be more sensitive to APAP-mediated liver failure.

## Results

### ASMase deficiency lowers the threshold for APAP hepatoxicity

**Figure 1:**
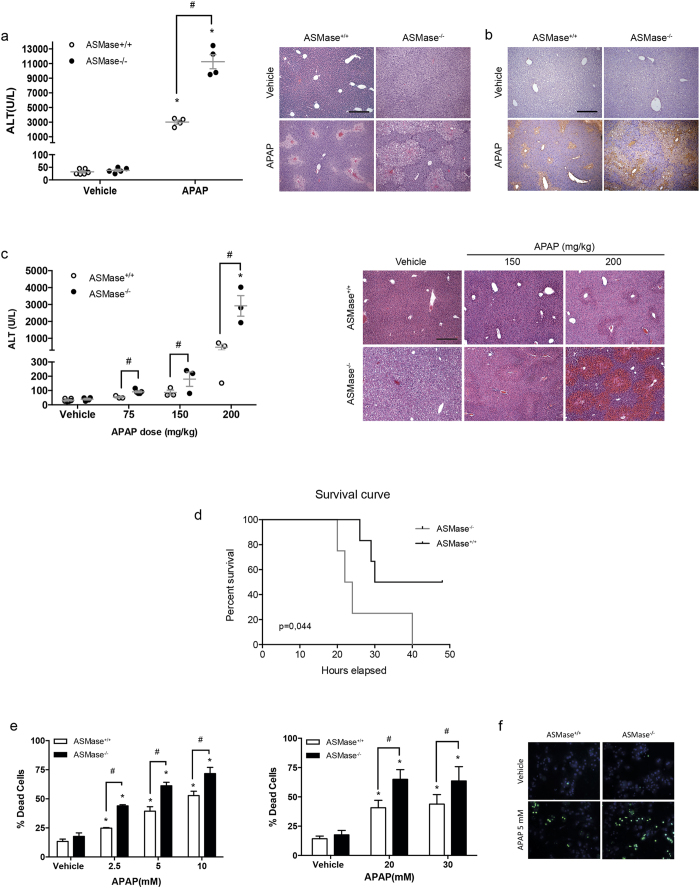
ASMase KO mice are more susceptible to APAP-induced liver toxicity (**a**) Serum ALT and H&E analyses of ASMase^+/+^ and ASMase^−/−^ mice 24 hours after APAP administration (i.p. 300 mg/Kg). (**b**) Liver samples were processed for TUNEL staining. (**c**) Serum ALT and H&E analyses of ASMase^+/+^ and ASMase^−/−^ mice 6 hours after treatment with different doses of APAP (i.p. 150 and 200 mg/Kg). (**d**) Survival rate of ASMase^+/+^ and ASMase^−/−^ mice at different time points after injection with a lethal dose of APAP (i.p. 600 mg/Kg) (n = 6 per group). (**e**) Trypan blue determination of cell viability of PMH from ASMase^+/+^ and ASMase^−/−^ mice after 24 hours exposure to different doses of APAP (2.5–10 mM). In some cases, PMH were pretreated for 2 hours with APAP (20 mM and 30 mM) and continued for 24 hours in the absence of APAP to determine cell viability. (**f**) Representative images of PMH stained with SytoxGreen-Höescht after incubation with 5 mM APAP. Data are expressed as mean ± SEM, n = 4–8. *p < 0.05 vs vehicle treated group. # p < 0.05 vs. ASMase^+/+^ mice. Scale bar represents 100 μm.

Serum ALT levels were elevated 24 h after APAP administration (300 mg/kg) in overnight fasted ASMase^+/+^ mice, but this increase was 3-fold higher in ASMase^−/−^ mice (Fig. 1a). H&E and TUNEL staining of liver sections following APAP treatment revealed increased liver injury in ASMase^−/−^ mice compared to ASMase^+/+^ littermates (Fig. 1a,b). The increased susceptibility of ASMase^−/−^ mice was maintained at 200 mg/Kg APAP dose and also observed at lower APAP doses (75–150 mg/kg), which do not cause damage in ASMase^+/+^ litermates (Fig. 1c). Importantly, the rate of hepatocyte regeneration as evidenced by PCNA and Ki67 staining was similar in both genotypes (Fig. S1), indicating that the susceptibility of ASMase^−/−^ mice to APAP is not due to impaired hepatocyte regeneration and liver repair. To test the impact of ASMase deficiency on APAP-induced mortality, mice fasted overnight were intraperitoneally injected with a lethal dose of APAP (600 mg/kg) and survival was monitored during the next 50 hours post-treatment. As seen, the mortality rate was higher for ASMase^−/−^ mice compared to ASMase^+/+^ mice (Fig. 1d).

We next investigated the susceptibility of primary mouse hepatocyte (PMH) to APAP treatment (2.5–10 mM). ASMase^−/−^ PMH showed increased cell death compared to ASMase^+/+^ PHM (Fig. 1e) as revealed by Sytox Green staining (Fig. 1f). Preincubation with higher doses of APAP (20 mM and 30 mM) for 2 hr followed by APAP removal also induced a higher rate of cell death in ASMase^−/−^ PMH than ASMase^+/+^ PMH (Fig. 1e). These data uncover that ASMase deficiency enhances the susceptibility to APAP treatment both *in vivo* and in PMH.

### APAP metabolism, GSH depletion and JNK activation are independent of ASMase

**Figure 2:**
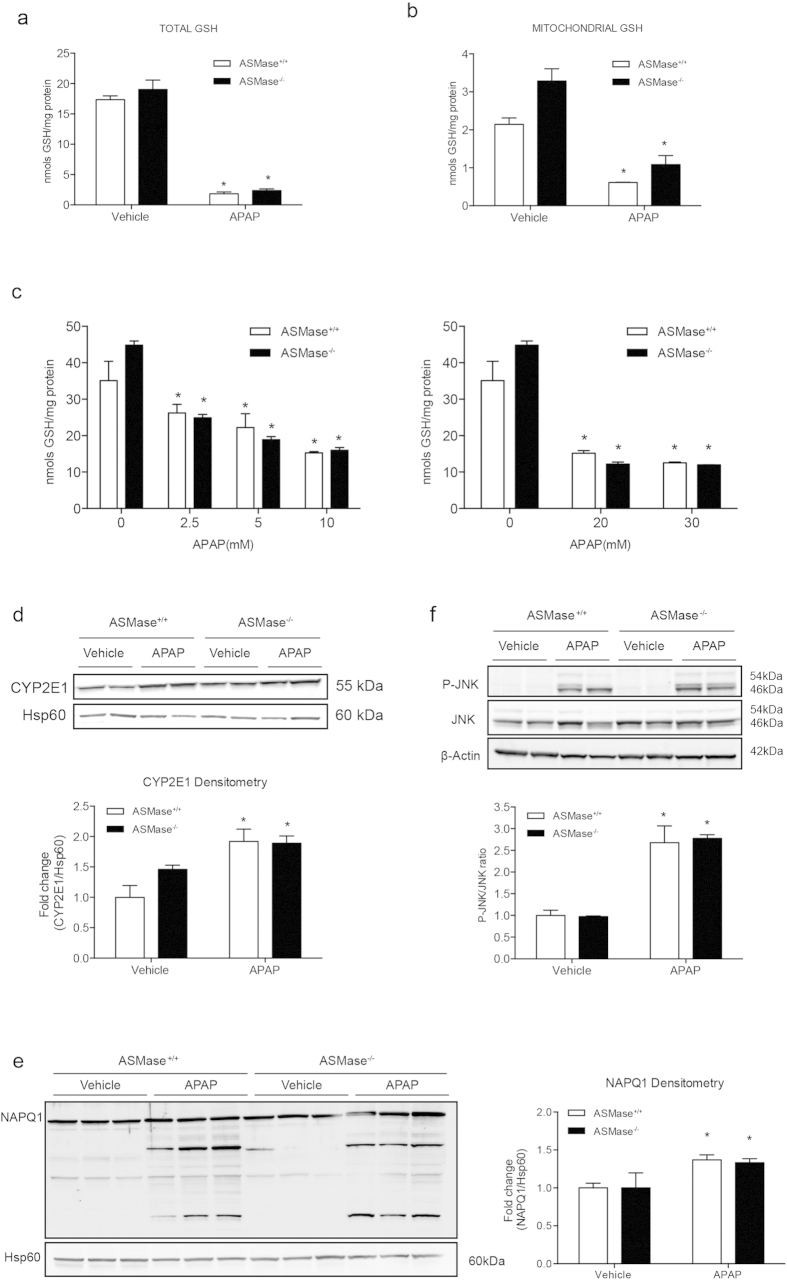
APAP metabolism and generation of toxic metabolites are independent of ASMase. (**a,b**) Hepatic total and mitochondrial GSH levels of ASMase^+/+^ and ASMase^−/−^ mice 2 hours after APAP injection. (**c**) Total hepatic GSH levels of ASMase^+/+^ and ASMase^−/−^ PMH 2 hours after APAP treatment at different doses. (**d–f**) CYP2E1 protein abundance, NAPQ1 adduct formation and phospho-JNK levels of mitochondrial samples of liver tissues harvested 2 h after APAP injection. Data are expressed as mean ± SEM (n = 3–6 mice) *p < 0.05 vs ASMase^+/+^ vehicle treated mice or PMH.

To investigate the molecular basis by which ASMase deficiency exacerbates APAP-induced hepatotoxicity, we analyzed predominant toxic mechanisms involved in APAP hepatotoxicity^6^,^24^. The levels of total and mitochondrial GSH of liver samples from mice treated with APAP (300 mg/kg, 2hr) decreased to a similar extent regardless of the genotype (Fig. 2a,b). The depletion of total GSH in PMH treated with various doses of APAP (2 hr) was similar between ASMase^+/+^ and ASMase^−/−^ PMH (Fig. 2c). Cytochrome P450 2E1 (CYP2E1), which is key in the generation of the hepatotoxic metabolite NAPQI^25^, was upregulated to a similar extent following APAP treatment in both types of mice (Fig. 2d). Moreover, NAPQI adduct formation after APAP treatment (2 hr) was also similar in both types of mice (Fig. 2e). Phosphorylated JNK (P-JNK), which has been documented to amplify APAP-induced mitochondrial oxidative stress and hepatotoxicity^3^,^24^, was activated to the same level in both genotypes after APAP administration (2 hr) (Fig. 2f). Similar levels of P-JNK were observed in PMH of ASMase^+/+^ and ASMase^−/−^ mice treated with 5 mM APAP for various times (Fig. S2a). Thus, these data discard a role for ASMase in APAP metabolism, GSH depletion and JNK activation.

### ASMase deficiency does not recruit additional mechanisms contributing to APAP hepatotoxicity

It has been described that NAPQI can bind to hepatic protein lysine residues and that the status of lysine acetylation regulates APAP hepatotoxicity^26^. The pattern of acetylated lysine residues was similar in ASMase^+/+^ and ASMase^−/−^ mice (Fig. S2b). Expression of connexin 32 (Cx32), a gap junction protein that regulates drug and APAP induced liver injury^27^, increased in total liver homogenates to a similar extent in both ASMase^+/+^ and ASMase^−/−^ mice (Fig. S2c). The levels of *Chop* mRNA induced by APAP were similar in both ASMase^+/+^ and ASMase^−/−^ mice (Fig. S2d). Moreover, the levels of nitrotyrosine protein adducts, which contribute to APAP hepatotoxicity^28^, were similar for both genotypes 6 h after APAP administration (Fig. S2e). Furthermore, although APAP causes lysosomal instability and cathepsin B release^16^ and ASMase^−/−^ mice exhibit cathepsin B overexpression^29^, Ca-Me064, a specific cathepsin B inhibitor, failed to prevent the sensitization of ASMase^−/−^ PMH to APAP treatment (not shown). These findings rule out additional toxic mechanisms in the susceptibility of ASMase^−/−^ mice to APAP hepatotoxicity.

### ASMase deficiency impairs APAP-induced fusion of mitochondria-containing autophagosomes with lysosomes

**Figure 3:**
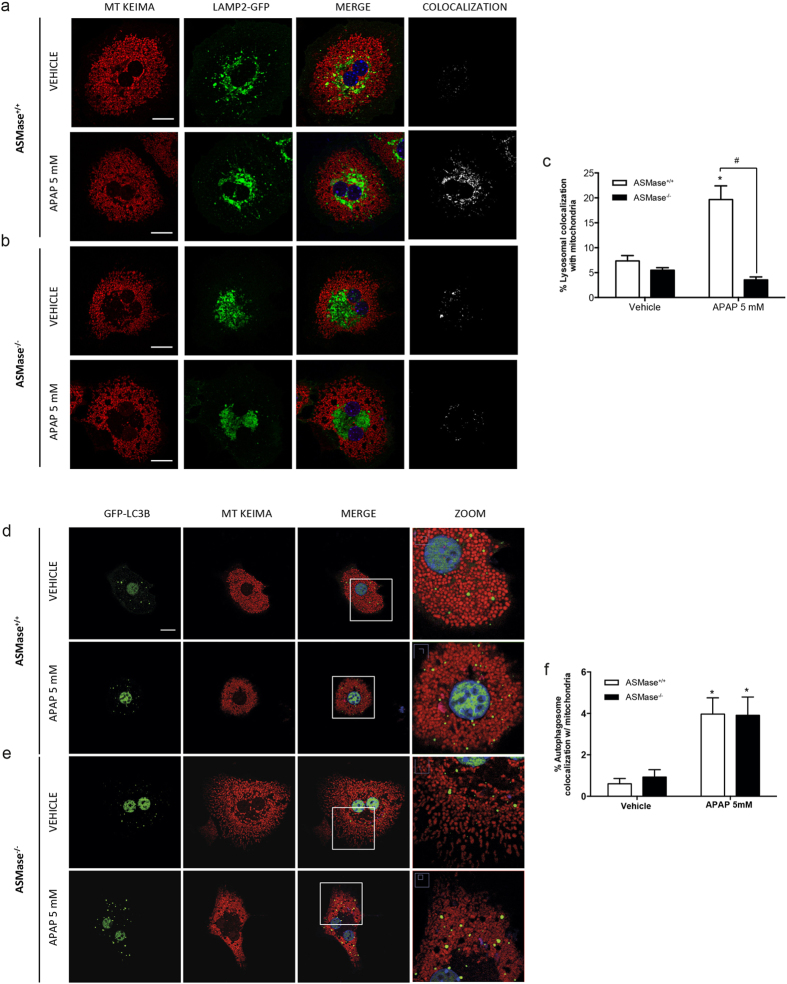
ASMase^−/−^ PMH exhibit decreased percentage of lysosomal colocalization with mitochondria (**a,b**) ASMase^+/+^ and ASMase^−/−^ PMH expressing LAMP2-GFP and mtKeima were treated with APAP (5 mM) for 3 hours and lysosomal colocalization with mitochondria was analysed by confocal imaging as white masks shown in the colocalization columns as described in Supplementary Methods. (**c**) 5 images per treatment of 3 different experiments were quantitated for colocalization of lysosomes with mitochondria. (**d–f**) ASMase^+/+^ and ASMase^−/−^ PMH expressing GFP-LC3B and mtKeima were treated with APAP (5 mM) for 6 hours to analyse and quantitate autophagosome colocalization with mitochondria (**f**) as described with (**c**) Data are expressed as mean ± SEM of 4 independent experiments. *p < 0.05 vs ASMase^+/+^ vehicle-treated PMH, #p<0.05 vs ASMase^+/+^ APAP. Scale bar represents 20 μm.

Although autophagy regulates APAP hepatotoxicity^11^,^12^,^30^ and ASMase deficiency impairs the fusion of autophagosomes with lysosomes^19^,^20^, the specific role of LC accumulation in mitophagy and APAP hepatotoxicity has not been previously characterized. We first confirmed that ASMase^−/−^ PMH exhibited increased free cholesterol levels revealed by filipin staining that colocalized with lysosomes (Fig. S3a) compared to ASMase^+/+^ PMH. Confocal imaging of PMH expressing LAMP-GFP to label lysosomes and mitochondria-targeted monomeric Keima red fluorescent protein (mtKeima) to label mitochondria^31^ revealed decreased lysosomal colocalization with mitochondria induced by APAP in ASMase^−/−^ PMH compared to ASMase^+/+^ PMH (Fig. 3a,c), suggesting that ASMase deficiency impaired APAP-induced fusion of mitochondria-containing autophagosomes with lysosomes. Moreover, we observed reduced mitochondrial DNA content in ASMase^+/+^ but not ASMase^−/−^ PMH (Fig. S4a) and APAP-induced ROS was higher in ASMase^−/−^ PMH than in ASMase^+/+^ PMH (Sup Fig. 4b), indicating persistent mitochondrial dysfunction following APAP in ASMase^−/−^ hepatocytes.

Next, we addressed whether the defect in mitophagy occurred at the level of formation of autophagosomes-containing mitochondria. Confocal imaging of PMH expressing GFP-LC3B to label autophagosomes and mtKeima to label mitochondria indicated a similar percentage of colocalization of autophagosomes with mitochondria after APAP regardless of the genotype (Fig. 3d–f). Although basal levels of Rab7 and the SNARE component Vamp8, which are known to regulate autophagosome fusion to late endosomes preceding transition from amphisomes to autolysosomes^32^, were higher in ASMase^−/−^ PMH than ASMase^+/+^ PMH, APAP-induced Rab7 expression was observed in ASMase^+/+^ PMH without effect on Vamp 8 (Fig. S5). These findings point that the defect in mitophagy by ASMase deficiency occurs at the level of fusion of autophagosomes-containing mitochondria with lysosomes. Although the increase in Rab7 and Vamp8, which are involved in an alternative pathway of autolysosome formation following the fusion of late endosomes with autophagosomes, may reflect a compensatory mechanism to correct fusion defects in ASMase^−/−^ PMH, this outcome may also indicate that the defect in the autophagosome-lysosome fusion is independent of amphisome formation and suggests that alterations in lysosomal membrane dynamics mediated by cholesterol accumulation can contribute to defects in autolysosome generation.

### Lysosomal cholesterol regulates APAP-mediated cell death and mitophagy

**Figure 4:**
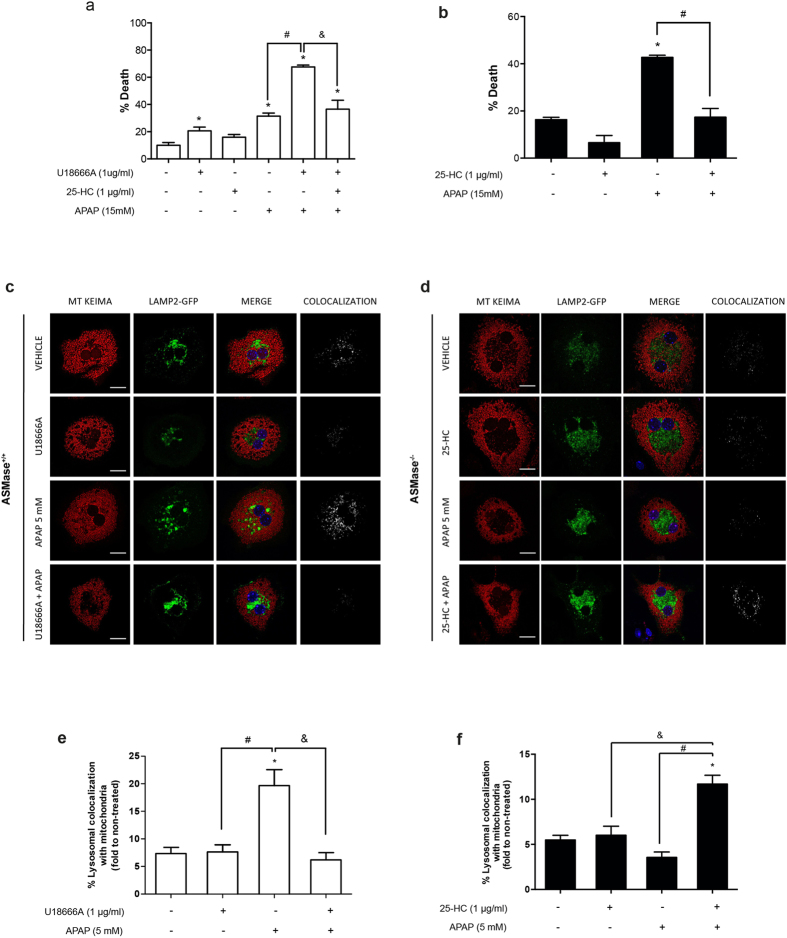
Effect of U18666A and 25-HC in lysosomal colocalization with mitochondria and APAP susceptibility (**a**) ASMase^+/+^ PMH were pretreated with U18666A with or without 25-HC for 12 hour and then incubated with APAP (15 mM) for 6 hours to determine cell viability by trypan blue exclusion. (**b**) Cell viability of ASMase^−/−^ PMH after incubation with APAP (15 mM) with or without 25-HC pretreatment. (**c,d**) ASMase^+/+^ and ASMase^−/−^ PMH expressing Lamp-GFP and mtKeima with or without pretreatment with U18666A or 25-HC were incubated with APAP (5 mM) for 3 hours to analyze lysosomal colocalization with mitochondria by confocal imaging. (**e,f**) 5 images per treatment of 3 different experiments were analyzed with Image J to assess the percentage of lysosomal colocalization with mitochondria. Data are expressed as mean ± SEM of 3 independent experiments. *p < 0.05 vs vehicle-treated group; #p < 0. 05 vs APAP-treated group and &p < 0. 05 vs APAP+U18666A treated group. Scale bar represents 20 μm.

Previous studies indicated that the cationic amphiphilic drug U18666A disrupts cholesterol trafficking and induces LC accumulation^33^,^34^. Hence, we examined the role of LC accumulation in APAP hepatotoxicity by analyzing whether treatment of wild type PMH with U18666A reproduces the sensitization to APAP seen in ASMase^−/−^ PMH. U18666A increased LC content determined by confocal staining with filipin (Fig. S3a,b) and sensitized to APAP-induced cell death (Fig. 4a). Furthermore, U18666A pretreatment abolished APAP-induced autolysosome formation in PMH expressing LAMP2-GFP and mt-Keima, as reflected by the decreased percentage of lysosomal colocalization with mitochondria (Fig. 4c,e).

To further confirm the role of LC accumulation in APAP hepatotoxicity, we addressed whether decreasing LC levels in ASMase^−/−^ PMH protects against APAP-mediated cell death. The oxysterol 25-HC, which acts as a ligand for liver X receptors and suppresses sterol synthesis, decreases cholesterol accumulation^33^,^34^. Moreover, previous findings indicated that 25-HC preferentially reduced the LC pool in Niemann Pick type C cells^35^. 25-HC pretreatment of ASMase^−/−^ PMH decreased LC accumulation as assessed by colocalization of filipin with LAMP2 immunofluorescence staining and the consequent analysis of filipin intensity (Fig. S3a,d). Moreover, 25-HC pretreatment protected ASMase^−/−^ PMH from APAP-induced cell death (Fig. 4b), and increased APAP-mediated percentage of lysosomes colocalizing with mitochondria, indicating improved mitophagy (Fig. 4d,f). Furthermore, 25-HC reduced LC levels in wild type PMH treated with U18666A (Fig. S3a,c) and protected againt APAP-mediated cell death (Fig. 4a). Importantly, *in vivo* treatment with 25-HC protected ASMase^−/−^ mice against APAP-induced liver injury with similar results seen in ASMase^+/+^ mice (Fig. S6). The protection of ASMase^−/−^ PMH or U18666A-treated ASMase^+/+^ PMH by 25-HC against APAP-induced cell death was abrogated by incubation with chloroquine to block autophagy (not shown).

**Figure 5:**
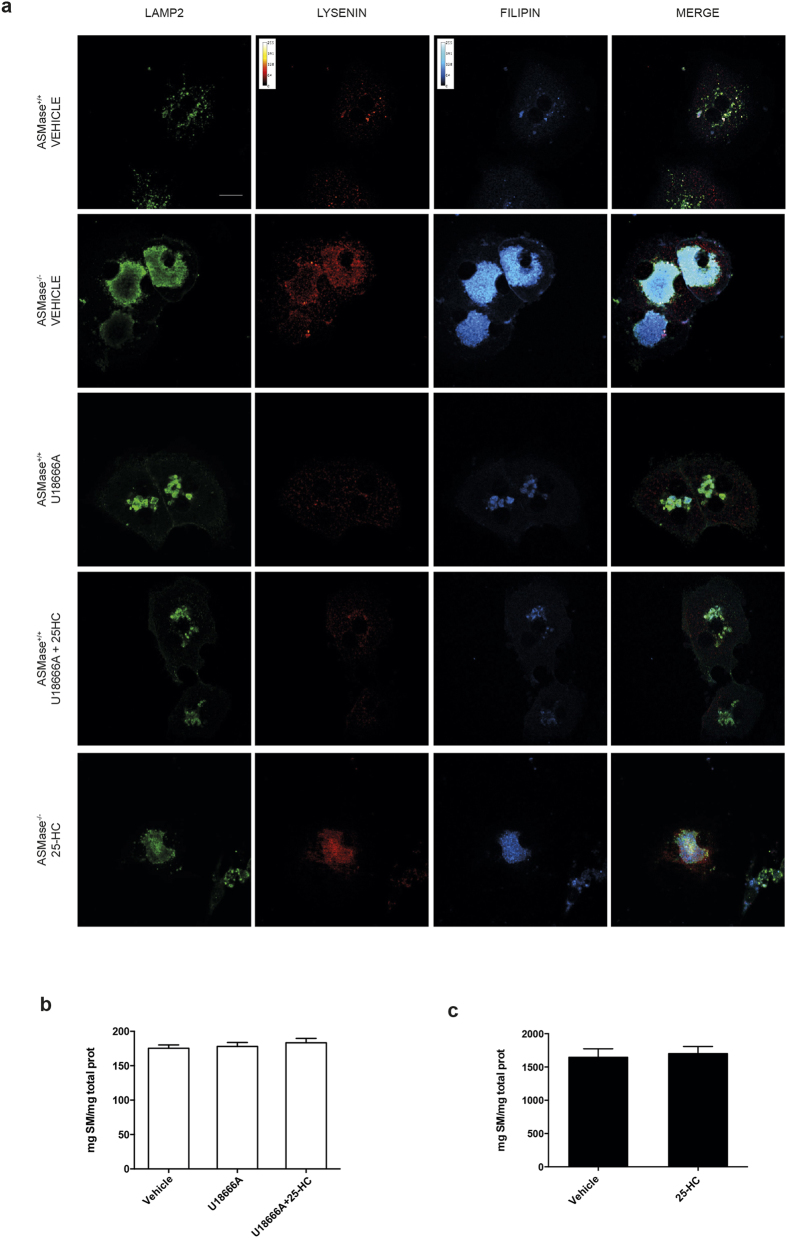
Effect of U18666A and 25-HC in the content and distribution of lysosomal sphingomyelin. (**a**) ASMase^+/+^ and ASMase^−/−^ PMH were treated overnight with U18666A (1 μg/ml), 25-HC (1 μg/ml) or both and lysosomal colocalization (Lamp2 staining) with cholesterol (filipin staining) and sphingomyelin (lysenin staining) was analysed by inmunofluorescence. (**b,c**) Total sphingomyelin levels in PMH following the different treatments were analysed with a colorimetric detection kit. Scale bar represents 20 μm.

We next addressed whether U18666A and 25-HC exerted any effect in sphingomyelin homeostasis in PMH. Compared to the increased filipin staining, indicative of higher free cholesterol content, total sphingomyelin levels determined by immunofluorescence with lysenin, a toxin that specifically recognizes sphingomyelin^36^ and colorimetric quantitation were unaltered in wild type PMH after U18666A exposure regardless of treatment with 25-HC (Fig. 5a,b). Furthermore, total sphingomyelin levels were 10-fold higher in ASMase^−/−^ PMH compared to wild type PMH, but remained unaltered following 25-HC pretreatment (Fig. 5a,c). Analyses of sphingomyelin areas colocalizing with lysosomes in ASMase^−/−^ hepatocytes discarded changes in lysosomal sphingomyelin content induced by 25-HC. These findings point to excess of LC rather than sphingomyelin as an important contributor in enhanced APAP hepatotoxicity.

### Human hepatocytes treated with amitriptyline exhibit LC accumulation, impairs mitophagy and APAP sensitization

**Figure 6:**
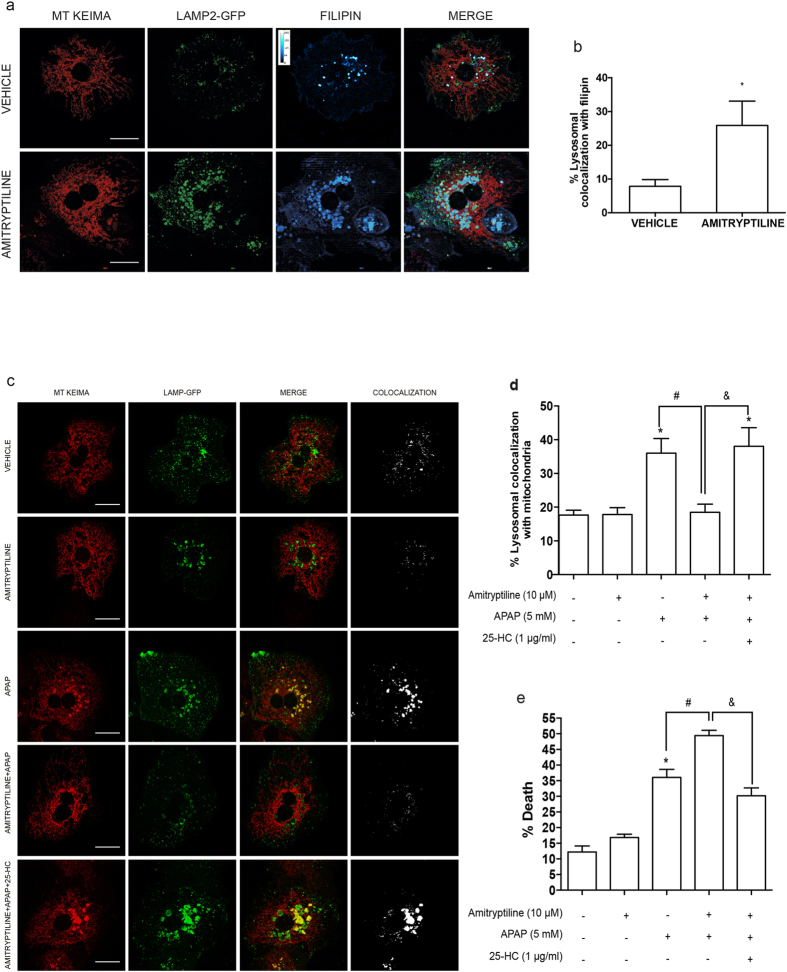
Human hepatocytes pretreated with amitriptyline exhibit increased LC accumulation, impaired mitophagy and sensitization to APAP-induced cell death. (**a**) Primary human hepatocytes expressing Lamp-GFP and mtKeima were overnight treated with amitriptyline (10 μM) and free cholesterol was stained with filipin. (**b**) 5 images per treatment were analyzed with Image J to assess the percentage of lysosomal colocalization with filipin. Images are representative of 3 independent experiments. Scale bar represents 20 μM. (**c,d**) Lysosomal colocalization with mitochondria of primary human hepatocytes expressing Lamp-GFP and mtKeima pretreated with Amitryptiline (10 μM) with or without 25-HC (1 μg/ml) for 12 hours following exposure to APAP (5 mM) for 3 hours. 5 images per treatment of 3 different experiments were analyzed with Image J to assess the percentage of lysosomal colocalization with mitochondria. (**e**) Cell viability was analysed in human hepatocytes pre-treated with amitryptiline with or withoug 25-HC (1 μg/ml) and exposed APAP 15 mM) for 6 hours. Data are expressed as mean ± SEM of 3 independent experiments. *p < 0.05 vs vehicle-treated group; #p < 0.05 vs APAP-treated group and &p < 0.05 vs APAP+Amitryptiline treated group. Scale bar represents 20 μm.

Amitriptyline inhibits ASMase by preventing its proteolytic processing^23^. We next examined whether ASMase inhibition by amitriptyline exerted cell autonomous effects in human hepatocytes following APAP exposure. As seen, human hepatocytes treated with amitriptyline, which inhibited ASMase activity by 80%, exhibited increased filipin staining and lysosomal proliferation, reflecting LC accumulation (Fig. 6a,b), in line with previous findings with imipramine^37^. While amitriptyline did not affect mitochondrial or lysosomal morphology in human hepatocytes, it impaired the colocalization of mtKeima with LAMP-GFP caused by APAP and sensitized to APAP-induced cell death (Fig. 6c,d). Moreover, treatment with 25-HC protected human hepatocytes against amitriptyline sensitization to APAP and restored colocalization of mitochondria-targeted mtKeima with LAMP-GFP, indicative of improved mitophagy (Fig. 6c,e). In line with these findings in human hepatocytes, amitriptyline elicited LC accumulation and APAP sensitization in PMH from wild type mice and these effects were prevented by 25-HC treatment (Figs S7 and S8).

### Prolonged treatment of mice with amitriptyline increased APAP-induced liver injury

**Figure 7:**
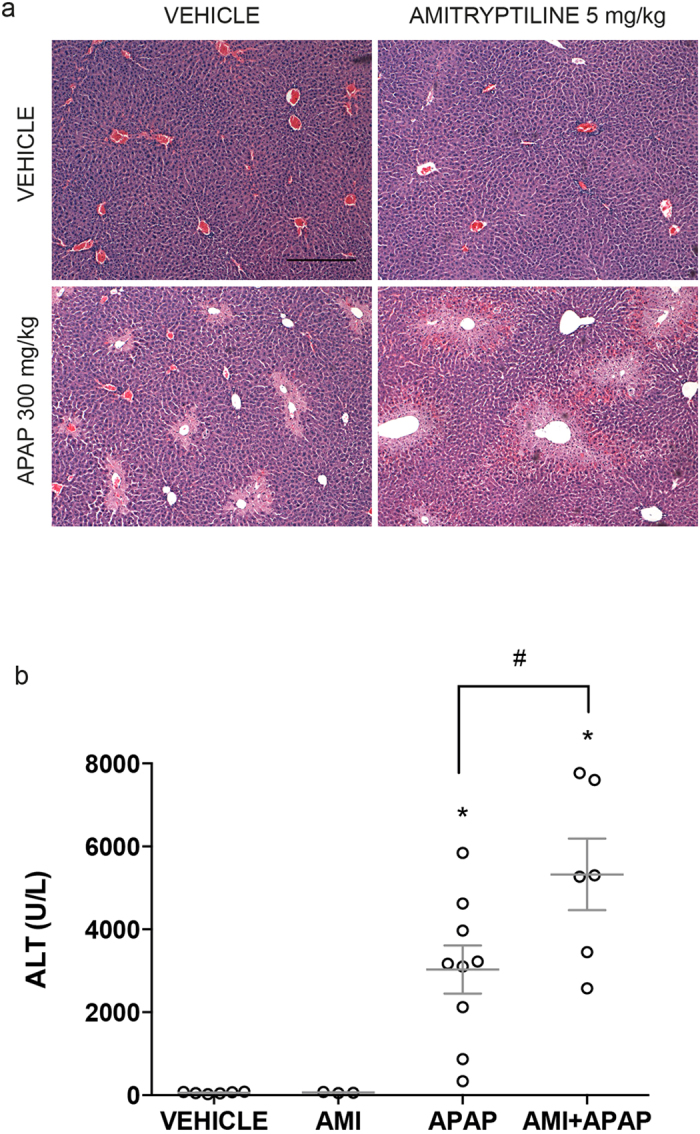
Prolonged treatment with amitriptyline sensitizes to APAP-mediated liver injury. (**a**) H&E staining of liver section from ASMase^+/+^ mice injected with amitriptyline (i.p. 5 mg/kg) or equal volume of saline as vehicle control for 5 days followed by APAP treatment (i.p. 300 mg/Kg) and sacrificed 6 hours later (**b**) Serum ALT levels measured 6 hours after APAP injection. Data are expressed as mean ± SEM; n = 3–9 mice per group. *p < 0.05 vs vehicle-treated group; #p < 0.05 vs APAP-treated group. Scale bar represents 100 μm.

To further address the relevance of the above findings in human or PMH hepatocytes, we examined the impact of treating mice with a therapeutic dose of amitriptyline (5mg/kg) for 5 days in sensitization to APAP. As seen, while protracted treatment with amitriptyline did not cause liver damage, it potentiated APAP-induced liver injury (Fig. 7a,b). Prolonged treatment with amitriptyline or 25-HC did not affect APAP metabolism as reflected by early GSH depletion, JNK activation or CYP2E1 expression (Fig. S9). These findings highlight the impact of chronic amitriptyline treatment in APAP hepatotoxicity in mice.

### Glucocerebrosidase inhibition increases LC, impairs mitophagy and sensitizes to APAP-induced cell death

We next examined whether a chemical model of GD triggered by CBE, which irreversibly inhibits glucocerebrosidase^22^, reproduces the phenotype of ASMase^−/−^ PMH and sensitization to APAP treatment. CBE inhibited glucocerebrosidase in wild type PMH by >80%. CBE increased filipin staining, which colocalized with lysosomes labeled with LAMP (Fig. S7) and impaired colocalization of mtKeima with LAMP-GFP and sensitized to APAP-induced cell death (Fig. S10). These findings further support the role of LC accumulation in APAP hepatotoxicity and imply that the sensitization to APAP may occur in Gaucher disease.

## Discussion

**Figure 8:**
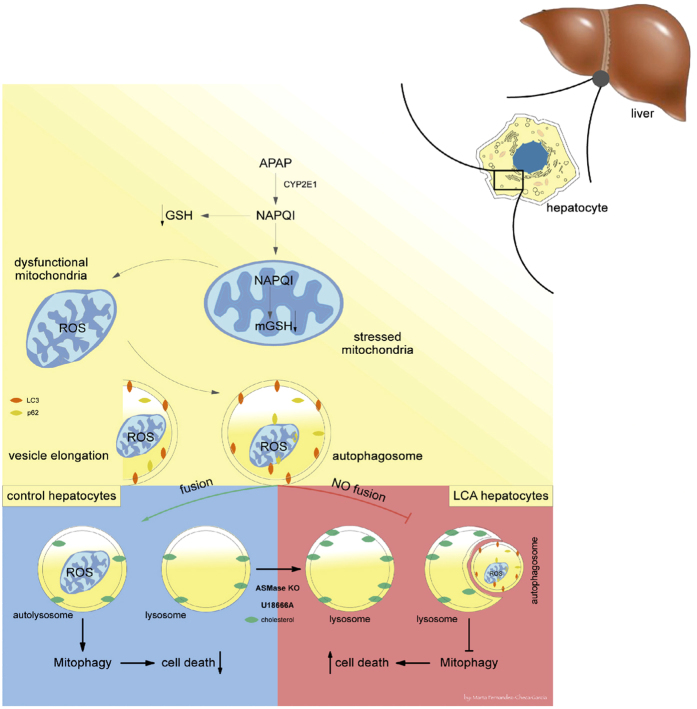
Schematic summary of the susceptibility of ASMase null mice to APAP. APAP metabolism and generation of toxic metabolites, including NAPQI and GSH depletion are independent of ASMase and cause mitochondria dysfunction. The elimination of stressed mitochondria in response to APAP is vital to limit liver injury. This process called mitophagy implies the fusion of autophagosomes containing stressed mitochondria with lysosomes for the degradation of mitochondria and ensures mitochondrial turnover. However, lysosomal cholesterol accumulation such in the case of NPA limits the fusion of autophagosomes with lysosomes, thus impairing mitophagy contributing to cell death.

The present study characterizes a novel mechanism involved in APAP hepatotoxicity. We provide evidence that LC accumulation sensitizes to APAP-mediated hepatocellular death and liver injury without increasing APAP metabolism or generation of cytotoxic metabolites (Fig. 8). ASMase^−/−^ mice exhibit increased liver injury in response to APAP, even at doses (75–200 mg/kg) that cause mild or no liver damage in ASMase^+/+^ mice. More importantly, ASMase deficiency determines increased mortality in response to a lethal dose of APAP. The primary consequence of ASMase deficiency in hepatocytes is the increase of lysosomal sphingomyelin levels. However, the accumulation of sphingomyelin can lead to a secondary increase in free cholesterol that is mainly confined to lysosomes, in line with findings in macrophages from ASMase^−/−^ mice^38^. The mechanism underlying LC accumulation secondary to increased sphingomyelin levels due to ASMase deficiency likely reflects the high affinity of sphingomyelin to bind cholesterol, which impairs the egress of cholesterol out of lysosomes^39^,^40^. Moreover, exposure of wild type macrophages to exogenous sphingomyelin reproduces the increased LC level reported in macrophages from ASMase^−/−^ mice^38^. Furthermore, sphingomyelin depletion in Chinese hamster ovary cells by exogenous sphingomyelinase blocks the proteolytic processing of SREBP-2 at site 1, while sphingomyelin enrichment facilitates the proteolytic processing of SREBP-2 to achieve an optimal ratio of sphingomyelin to cholesterol in membrane bilayers^41^. Thus, the relationship between ASMase activity and cholesterol appears to be regulated by sphingomyelin homeostasis.

Consistent with previous findings in murine hepatoma Hepa1c1c7 cell treated with the cationic amphiphilic drug imipramine^37^, we observed that amitriptyline, a widely used tricyclic antidepressant that inhibits ASMase^23^, induces LC accumulation, impairs colocalization of mitochondria with lysosomes and increased APAP-mediated cell death in human hepatocytes and wild type PMH. Furthermore, U18666A, a drug that reproduces *in vitro* the NPC phenotype, causes LC accumulation in wild type hepatocytes and sensitizes to APAP hepatotoxicity. Moreover, we also show that accumulation of glucosylceramide by CBE, which triggers GD^22^, increased filipin staining in lysosomes indicative of LC accumulation, in agreement with previous findings^42^, and results in the subsequent sensitization to APAP-mediated cell death. Thus, regardless of the mechanism (ASMase deficiency or treatment with U18666A or CBE) LC accumulation emerges as a key factor in APAP hepatotoxicity.

To estimate the relative contribution of LC accumulation versus increased lysosomal sphingomyelin levels in the sensitization to APAP hepatotoxicity in ASMase^−/−^ mice, we examined the effect of the oxysterol 25-HC, which regulates cholesterol homeostasis and relieves LC accumulation in NPC cells^34^,^35^. Treatment of hepatocytes from ASMase^−/−^ mice with 25-HC reduced the level of LC accumulation and protected against APAP hepatotoxicity; moreover 25-HC rescued ASMase^−/−^ mice from APAP-mediated liver injury. The protective effect of 25-HC was also observed in wild type hepatocytes following U18666A exposure resulting in decreased LC accumulation and APAP-induced cell death. Importantly, neither 25-HC nor U18666A changed the total levels of sphingomyelin or its lysosomal distribution in ASMase^−/−^ or ASMase^+/+^ hepatocytes, respectively. While these data discard a role for lysosomal sphingomyelin accumulation in the sensitization to APAP, we cannot rule out the possibility that other glycosphingolipids accumulating in endolysosomes in NPA may contribute to APAP sensitization.

Since autophagy has emerged recently as an important mechanism that protects against APAP hepatotoxicity^12^,^14^, potentially by clearing defective, ROS-producing mitochondria, we examined the formation of autophagosomes and autolysosomes containing mitochondria, as an estimation of early and late mitophagy, respectively. Compared to ASMase^+/+^ hepatocytes, ASMase deficiency decreases the percentage of lysosomes colocalizing with mitochondria without changing the rate of autophagosomes that colocalize with mitochondria, suggesting a defect in the fusion of autophagosomes with lysosomes (Fig. 8). As cholesterol is a critical determinant of the membrane’s physical properties^43^, the increase in lysosomal cholesterol likely decreases lysosomal membrane dynamics and reduces the transition from liquid-ordered to liquid-disordered phases, contributing to the impairment of the fusion of lysosomes with autophagasosomes containing mitochondria. The link between ASMase deficiency and impaired autophagy has been described not only in hepatocytes but also in mouse coronary arterial smooth muscle cells, brain from ASMase^−/−^ mice as well as in fibroblasts from patients with NPA and together point to an emerging role for ASMase in the regulation of autophagy^18^,^19^,^20^. The defect in autophagy in brain from ASMase^−/−^ mice or fibroblasts from NPA patients is intriguing as it was suggested that lysosomal sphingomyelin was the culprit. Incubation of NPA fibroblasts with methyl-β-cyclodextrin had no effect on LC3-II levels despite this response was associated with a mild reduction of total cholesterol levels by 16%^44^. In contrast to the moderate effect of methyl-β-cyclodextrin, we observe that 25-HC substantially decrease filipin staining (30–50%) in hepatocytes from ASMase^−/−^ mice or after treatment with U18666A without a change in total sphingomyelin levels. Whether specific experimental conditions, the type of cell used or approach to target cholesterol contribute to the differential outcome remains to be explored. Thus, our findings point to LC accumulation as an important factor determining sensitization to APAP hepatotoxicity by impairing fusion of lysosomes with autophagosomes (Fig. 8).

The present findings may have clinical implications. Factors that increase the risk of therapeutic misadventure from APAP use remain to be fully identified. Recent work using the FDA Adverse Event Reporting System identified some comedications influencing the clinical outcome of APAP-associated liver injury by modulating liver injury/repair equilibrium^45^. Among identified medications included sympathetic stimulants, such as alpha adrenoreceptor agonists. While amitriptyline is a tricyclic antidepressant its analgesic effects require alpha 2A adrenoreceptor^46^, suggesting that it functions as a alpha 2A adrenoreceptor agonist. Our findings show that amitriptyline exerts cell-autonomous effects in human hepatocyes resulting in LC accumulation and sensitization to APAP hepatotoxicity, and suggest that amytriptyline may lower the threshold for APAP susceptibility. In line with this possibility, it has been shown that administration of desipramine, another tricyclic antidepressant functionally related to amitriptyline, potentiated APAP hepatotoxicity^47^ without effect in the adsorption of APAP nor its excretion as APAP-mercapturic acid in in human volunteers^48^. Furthermore, desipramine treatment increased APAP-induced mortality in mice^47^, in agreement with our findings using amitriptyline. Interestingly, our data also indicate that 25-HC protect against APAP hepatotoxicity by decreasing LC accumulation. Therefore, the present results strongly suggest that ASMase deficiency may stand as a risk factor in the sensitization not only to APAP overdose but also to low doses (75–150 mg/K) that do not cause liver damage in ASMase^+/+^ littermates. Overall these findings may further stimulate clinical research to uncover unrecognized clinically used drugs that sensitize to APAP hepatotoxicity and to explore the potential benefits of 25-HC in ameliorating APAP-mediated liver injury.

## Materials and Methods

### Mice and treatments

ASMase^−/−^ mice (in C57BL/6J strain) and their control ASMase^+/+^ littermates (8–10 weeks old) were generated using heterozygous breeding pairs and genotyped as previously described^49^,^50^. All the experimental protocols used were approved and performed in accordance with the Animal Care Committee of the Hospital Clinic-Universidad de Barcelona and the USC Keck School of Medicine, including the use of ASMase null mice, isolation of primary mouse hepatocytes and mitochondria. The animals were fasted overnight (water was available) prior to experiments. APAP (Sigma), dissolved in warm PBS (55 °C) and cooled to 37 °C before injection, was administered intraperitoneally to mice (150–300 mg/kg). For the survival curve, higher dose of APAP was administered (600 mg/kg). In some cases, mice were treated with a subcutaneous injection of 25-HC (30 mg/kg) disolved at 6 mg/ml in a 45% 2-propyl-β-cyclodextrin as described^51^.

### Hepatocyte isolation and cell culture

Hepatocytes from ASMase^−/−^ mice and ASMase^+/+^ mice were isolated by collagenase perfusion with flow rate of 8.5 ml/min and were cultured on dishes coated with rat tail collagen as described previously^51^,^52^. Hepatocytes were treated with APAP up to 10 mM for 24 hr. In some cases, hepatocytes were treated with 20 mM and 30 mM APAP for 2 hr, then washed to remove APAP, and incubated for 24 hr to determine cell viability. In some experiments, hepatocytes were treated overnight with U18666A (1 μg/ml, Calbiochem) or 25-HC (1 μg/ml, Sigma) prior to APAP treatment (15 mM). Moreover, hepatocytes were exposed to amitriptyline or CBE to inhibit ASMase or glucocerebrosidase, respectively, and then challenged with APAP (15 mM).

### Human Hepatocytes

Cryopreserved human hepatocytes were purchased from Biopredic International. Hepatocytes were thawed and plated as described by the company. Briefly, hepatocytes were thawed using Leibovitz’smL15 medium and suspended in enough volume of seeding medium (supplemented Williams E GlutaMAX, Life Technologies) to plate 0.38 × 10^6^ cells/well in a 24-well plate previously coated with rat-tail collagen (Life Technologies). After an overnight incubation in serum containing medium, the seeding medium was replaced with warm maintenance medium (Williams E GlutaMAX without fetal bovine serum, Life Technologies). Hepatocytes were treated overnight either with amitryptiline (10 μM) or amitryptiline and 25-HC (1 μg/ml, Sigma) prior to APAP exposure at 15 mM for 6 hours to determine cell viability. For the confocal microscopy experiments, hepatocytes were previously transduced with Adenovirus LAMP-GFP and Adenovirus mtKeima as described in detail in Supplementary Methods section. This section also describes Hepatocyte isolation, Adenoviral treatment, and *in vivo* treatment with amitriptyline and 25-HC.

### Cell viability

Cell viability was determined by trypan blue exclusion (0,2%, Sigma) or by double stainning with 8 μg/ml Hoechst 33258 (Molecular Probes, Life Technology) and 1 μM Sytox green (Molecular Probes, Life Technology). Briefly, cells were trypsinized and resuspended in media. A small aliquot was diluted with Trypan Blue and cells were counted in a Neubauer Chamber. Each sample was analyzed twice. For the double staining method, Hoechst 33258, a nucleic acid stain that binds dsDNA of both live and dead cells was added to the cells for 15 min. Sytox green, a nucleic acid dye that stains dead cells with compromised membranes was added just before analysis. Culture dishes were observed under an OLYMPUS fluorescent microscope. Quantitation of total and necrotic cells (Sytox green positive) was performed as previously described by counting >1,000 cells in 10 different fields^53^.

### *In vivo* treatment with amitryptiline and 25-HC

C57BL/6J wild-type mice (8 weeks) were injected intraperitoneally with amitryptiline (5 mg/kg, dissolved in saline, Sigma Aldrich,) once a day during five days. The fourth day, animals were overnight fasted, and one hour after the last administration of amitryptiline, animals were injected intraperitoneally with APAP (300 mg/kg) and sacrificed 6 hours later. Serum and liver sections were collected for further analyses. A similar protocol was used for 25-HC administration (30 mg/Kg). 25-HC was dissolved in (2-hydroxypropyl)-β-cyclodextrin (Sigma Aldrich) at 45% (w/v). One hour after the last 25-HC administration animals were injected intraperitoneally with APAP (300 mg/kg) and sacrificed 6 hours later. Serum and liver sections were collected for further analyses.

### Adenoviral generation

Lamp-GFP and mtKeima adenovirus were generated using the ViraPower Adenoviral Expression System (Invitrogen) according to the manufacturer’s instructions. Briefly, High Fidelity Pfx50 DNA polymerase (Invitrogen) was used to amplify the coding sequences of interest with flanking BP sites. The coding sequence for Lamp-GFP was amplified from the Lamp1-GFP plasmid^54^ (Addgene #34831) and the coding sequence for mitochondrially-targeted monomeric Keima Red was amplified from the mt-mKeima/pIND(SP1) plasmid^55^, a generous gift from Dr. Miyawaki (RIKEN Brain Science Institute). Purified BP fragments containing the coding sequences were recombined into the pDONR221 vector using Gateway BP clonase II to obtain Entry clones. Lamp-GFP and mtKeimaRed were transferred from the Entry clones into the pAd/CMV/V5-DEST vector (Invitrogen) using the Gateway LR Clonase II enzyme mix. Recombinant adenoviral purified plasmid was digested with PACI and transfected into 293A cells using Lipofectamine 2000 reagent (Invitrogen). The 293A cells were maintained until 70% cytopathic effect was observed, tipically 5–10 days post-transfection. Cells and media were collected and subjected to three freeze/thaw cycles. The cell debris was pelleted at 3000 rpm for 15 min, and the supernatant was used to transduce a new set of 293A cells for adenovirus amplification. Afterwards, cells were lysed as above, and supernatant was aliquoted and titered using the AdenoX Rapid titer kit (Clontech).

### Adenoviral transduction in primary hepatocytes

Freshly isolated primary hepatocytes were plated on collagen-coated coverslips on 12 well plates at 125000 cells per well. After 3 hours of incubation in F-12/DMEM media with 10% FBS supplemented with penicillin/streptomycin, primary hepatocytes were switched with same media without FBS and transduced with Adenovirus Lamp-GFP and Adenovirus mtKeima at MOI of 10 and 25, respectively. After overnight incubation media was changed and cell were incubated with APAP for 2–6 hours and analyzed by confocal microscopy.

### Mitophagy determination by laser confocal imaging

Human or primary mouse hepatocytes expressing LAMP-GFP and mtKeima were analyzed by confocal imaging. Cells were fixed with 4% paraformaldehyde and mounted on glass slides with Fluoroshield and stained with DAPI. In some cases, coverslips were incubated with filipin (250 μg/ml, Sigma) 1 h at room temperature, washed twice with PBS and mounted with Dako fluorescent mounting medium (Dako Diagnostico SA, Spain). The slides were visualized in a Leica SPE confocal laser-scanning microscope. Percentage of total lysosomal mass containing mitochondria was analyzed with the *Colocalization nBits nimages* plugin (Confocal Miscroscopy Unit, Facultad de Medicina, Universidad de Barcelona) in the Image J Software in 5 consecutive images of each experimental condition. This plug-in sofware highlights the colocalized points of two images of 32-bits, and returns the integrated densities of total green (higher than the threshold) and the green from colocalized points and total red (higher than the threshold) and red from colocalized points. Two points are considered as colocalized if their respective intensities are strictly higher than the threshold of their channels and if their ratio of intensity is strictly higher than the ratio setting value, which have been defined at 50%. Percentage of green colocalization with red or red colocalization with green is calculated as the ratio of green colocalized points divided by total green multiplied per 100 or as the ratio of red colocalized points divided by total red multiplied per 100, respectively.

### Sphingomyelin measurement and immunofluorescence

Sphingomyelin levels in primary mouse hepatocytes extracts were measured with a colorimetric commercial kit (Cayman). Lipid extraction from 1 mg of total protein was performed with methanol:chloroform (1:2). Lipid extracts were dried and resuspended in a 100 μl of the kit´s detergent solution and incubated 10 min at 50 °C. 10 μl of this final solution was used to measure sphingomyelin content, which was normalized by protein concentration. To stain sphingomyelin in primary hepatocytes, lysenin (PeptaNova), a sphingomyelin-specific binding protein^36^, was added during the overnight incubation of the primary antibody. Coverslips were washed and incubated with lysenin rabbit-antisera (PeptaNova) during 2 hours at room temperature and then, cells were washed twice with PBS and incubated with secondary antibody (ant-rabbit IgG antibody conjugated with Alexa Fluor 594; Molecular Probes). Coverslips were processed as above and samples were visualized by laser-scanning microscopy (Alexa 594 λ_ex_594 nm ; λ_em_590–617 nm).

### Statistical Analysis

Results were expressed as mean ±SEM. Statistical significance of mean values was assessed using Student t-test and one-way ANOVA followed by Bonferroni post-test. Statistics were performed using GraphPad Prism 6 software. p ≤ 0.05 was defined as statistically significant.

## Additional Information

**How to cite this article**: Baulies, A. *et al.* Lysosomal Cholesterol Accumulation Sensitizes To Acetaminophen Hepatotoxicity by Impairing Mitophagy. *Sci. Rep.* **5**, 18017; doi: 10.1038/srep18017 (2015).

## Supplementary Material

Supplementary Information
