# Supplementary material for: Lysosomal Cholesterol Accumulation Sensitizes To Acetaminophen Hepatotoxicity by Impairing Mitophagy
Source: Sci Rep. 2015 Dec 11;5:18017. doi: 10.1038/srep18017 (PMC4676017; doi:10.1038/srep18017)
Supplement: Supplementary Information [file srep18017-s1.pdf]

SUPPLEMENTARY INFORMATION

LYSOSOMAL CHOLESTEROL ACCUMULATION SENSITIZES TO ACETAMINOPHEN HEPATOTOXICITY BY IMPAIRING MITOPHAGY

Anna Baulies[1,2], Vicent Ribas[1,2], Susana Nuñez[1,2], Sandra Torres[1,2], Cristina Alarcon-Vila[1,2], Laura Martínez[1,2], Jo Suda[3], Maria D. Ybanez[3], Neil Kaplowitz[3], Carmen García-Ruiz[1,2,4], Jose C. Fernández-Checa[1,2,4]

SUPPLEMENTARY METHODS

METHODS

mtDNA Quantification by Quantitative Real-Time PCR

Quantitative PCR was performed in duplicate with 5ng of DNA using 10 µM of each primer (mtDNA specific PCR (ND1**:** NADH dehydrogenase subunit 1; forward: CCC ATT CGC GTT ATT CTT; reverse: AAG TTG ATC GTA ACG GAA GC); and nuclear specific PCR (LPL 225 bp fragment: lipoprotein lipase; forward: GGA TGG ACG GTA AGA GTG ATT C; reverse: ATC CAA GGG TAG CAG ACA GGT)) and Power SYBRR Green PCR Master Mix (Applied Biosystems) in real-time PCR system (iCycler Thermal Cycler, Bio-Rad) with a program of 20 minutes at 95°C, followed by 50 to 55 cycles of 15 seconds at 95°C, 20 seconds at 58°C and 20 seconds at 72°C. Single-product amplification was verified by an integrated post-run melting curve analysis. Results were calculated from the difference in threshold cycle (RCT) values for mtDNA and nuclear specific amplification.

Biochemical measurements

To determine liver injury, serum ALT and AST were determined by standard procedures of the Clinical Chemistry laboratory of Hospital Clinic de Barcelona. GSH levels in homogenates, mitochondria or cytosols were determined by the recycling method.

Hematoxylin and Eosin and TUNEL staining

For hematoxylin and eosin staining, fresh liver samples were collected and fixed in formalin for 48h. Fixed livers were embedded in paraffin and placed in blocks and 7 μm slices were cut on a microtome. For TUNEL staining, paraffin-embedded liver tissue was cut into slices of 4 μm in thickness and fixed to glass slides. The TUNEL assay was performed by incubation of the slices with 2 μg/ml proteinase K (Sigma-Aldrich) in 10 mM Tris- HCl buffer containing 5 mM EDTA at a pH of 7.4. Sections were then labeled following the instructions of a commercial Kit (In situ Cell Death Detection Kit; Roche Diagnostics), stained with 3,3′-diaminobenzidine (Roche Diagnostics), and counterstained with hematoxylin and eosin.

Immunohistochemical Staining

Paraffin molds containing liver sections were cut into 5-μm sections and mounted on HistoGrip-coated slides. The sections were deparaffinized in xylene and dehydrated in a graded alcohol series. When necessary, endogenous peroxidase (3% H_2O_2) and endogenous avidin and biotin were blocked. Slides were incubated with primary antibody (PCNA, Santa Cruz or Ki67, AbCam) overnight in a wet chamber at 4°C. After a rinse with 1× PBS, the slides were incubated with a biotinylated antibody for 45 minutes in a wet chamber and developed with an ABC Kit with peroxidase substrate

(diaminobenzidine) and peroxidase buffer. After the slides were rinsed with tap water, they were counterstained with hematoxylin and mounted with Aquatex.

Mitochondria preparation

Livers were excised and homogenized in medium A (225 mM Mannitol; 75 mM Sucrose; 0,1 mM EGTA; 1 mg/ml FFA-BSA and 10 mM Hepes-KOH, pH 7.4). The homogenate was centrifuged at 700 g, 15 min at 4 ˚C, the pellet removed, and the supernatant centrifuged at 10.000 g, 15 min at 4˚C. After this centrifugation, the supernatant was discarded and the pellet containing the mitochondrial pellet was resuspended in buffer B (395 mM Sucrose; 0,1 mM EGTA and 10 mM Hepes-KOH, pH 7.4). The resuspended pellet was added to the top of a Percoll gradient (3 ml of 60% Percoll (v/v) and 7 ml of 26% Percoll (v/v) with buffer B) and was ultracentrifuged at 15.000 rpm, 35 min at 4˚C. Mitochondria was collected from the middle of the two Percoll layers and transferred to another tube where was resuspended with buffer B and centrifuged at 10.000 g, 15 min at 4˚C, to remove excess Percoll. Supernatant was discarded and the pellet containing mitochondria was resuspended with 350uL of Buffer A.

Immunofluorescence

Freshly isolated primary hepatocytes were plated on collagen-coated coverslips on 12 well plates at a 125000 cells per well. After the different treatments, cells were fixed with 4% paraformaldehyde (PFA) at room temperature and washed twice with PBS before proceeding with next steps. Then, cells were blocked and permeabilized for 30 min at room temperature with blocking buffer (PBS-0.5% glycine; 1% FFA-BSA; 0.025% saponinin) and incubated with Lamp2 (AbCam) primary antibody overnight. Cells were washed twice and then incubated with the secondary antibody (anti-rat IgG

antibody conjugated with Alexa Fluor 488; Molecular Probes) 1h at room temperature. In some cases, filipin (Sigma Aldrich, 250µg/ml) was added to stain free cholesterol during the secondary antibody. Coverslips were washed with PBS and mounted on glass slides with Dako fluorescent mounting medium. Samples were visualized in the Leica SPE confocal laser-scanning microscope (Filipin λ_{ex}340-380nm ; λ_{em}385-470nm. Alexa 488 λ_{ex}488nm ; λ_{em}495-519nm).

Western blotting

To analyze liver samples by western blotting, 100mg of liver were homogenized in pestle homogenizer with 1ml of homobuffer supplemented with an antiprotease cocktail (Complete, Roche). Liver homogenates were diluted 4 times in RIPA lysis buffer supplemented with antiproteases and antiphosphatases (PhosSTOP, Roche). Samples were incubated 10 min at 4ºC, vortexed and spin down 5 min at 10.000 rpm. All supernatants were collected and quantified for protein concentration using Bradford reagent (Bio-Rad). From 20 to 80µg of liver lysates was run in polyacrylamide gels. To analyze the mitochondrial fraction, isolated mitochondria resuspended in buffer A were diluted 4 times in RIPA lysis buffer. For the analysis of cultured cells by western blotting, media was removed and cells were washed twice with PBS and scrapped with RIPA lysis buffer supplemented with antiprotease and antiphophatase cocktail at 4ºC. Samples were incubated 10 min at 4ºC, vortexed and spin down 5 min at 10.000 rpm. 5% BSA solution in TBST was used to block the nitrocellulose blotting membranes (Amersham). Membranes were incubated with primary antibodies against P-JNK (Cell Signaling), JNK (Cell Signaling), CYP2E1 (AbCam), C-32 (Invitrogen), PINK1 (AbCam), Parkin (AbCam), VDAC (Millipore), Hsp60 (AbCam), TOM20 (Invitrogen) or β-actin (Sigma). NAPQI protein adducts antiserum was provided by Drs. Hinson and James from the University of Arkansas. Membranes were incubated for 45min with

HRP-conjugated secondary antibodies and developed with Pierce ECL Western Blotting Substrate (Thermo Scientific). Images were collected with the image-capturing instrument, LAS4000 (GE Healthcare) and analyzed with Image J free software.

RNA and DNA isolation

Total RNA or DNA from mouse and primary mouse hepatocytes were isolated with TRIzol® Reagent (Invitrogen). After homogenizing the sample with TRIzol®, chloroform was added, and the homogenate was allowed to separate into a clear upper aqueous layer (containing RNA), an interphase, and a red lower organic layer (containing the DNA and proteins). RNA was precipitated from the aqueous layer with isopropanol. Isopropanol was removed and the RNA pellet was air-dryed for 10min. RNA pellet was resuspended in RNase-free water and incubated at least 30min at 4ºC to allow complete resuspension. RNA was quantified and purity was checked measuring the sample absorbance at 260nm and 280nm with the NanoDrop spectrophotometer (260/280 ratio between 1.7-2.0). To isolate DNA, any remaining aqueous phase from the red organic layer was removed carefully to ensure the quality of the isolated DNA. Absolute ethanol was added per 1 mL TRIzol® used for the initial homogenization and tubes were inverted for mixing and incubated at room temperature during 3 minutes. Samples were centrifuged at 2000 x g for 5 minutes and the supernatant was removed to proceed with the washes. 1 mL of sodium citrate/ ethanol solution (0,1M sodium citrate in 10% ethanol, pH 8,5) was added to the pellet per 1 mL of TRIzol® used for the initial homogenization. Samples were incubated 30 min at room temperature and the wash out step was repeated twice. 75% ethanol was added in the last purification step and after a 20 min incubation, pellets were air dried and resuspended with buffer AE (10 mM Tris-HCl, 0,5 mM EDTA, pH 9). DNA was quantified and purity was checked

measuring the sample absorbance at 260nm and 280nm with the NanoDrop spectrophotometer (260/280 ratio between 1.6-1.8).

Real-Time PCR

Real-time PCR was performed using the iScript™ One-Step RT-PCR Kit with SYBR® Green (Bio-Rad) following the manufacturer instructions. Briefly, 20 ng of total RNA, 600 µmol/l of primers, and 12.5 µl of 2X Reaction Mix were incubated in 25 µl at 50 °C for 10 min and 95 °C for 5 min, followed by 45 cycles of 95 °C for 10 s, 56 °C for 30 s, and 72 ºC for 30 s. Each reaction was run in duplicate and the threshold (CT) values for each mRNA were subtracted from that of β-Actin mRNA, averaged and converted from log-linear to linear term. The primer sequences used were as follows:

CHOP forward 5’-CCACCACACCTGAAAGCAGAA-3’,

reverse 5’-AGGTGAAAGGCAGGGACTCA-3’

β-Actin; forward 5’-GACGGCCAGGTCATCACTAT-3’,

reverse 5’-CGGATGTCAACGTCACACTT-3’.

SUPPLEMENTARY FIGURE LEGENDS

Figure S1. Hepatocyte proliferation after APAP treatment.

Representative images of (a) PCNA and (b) ki67 staining in liver sections of $ASMase^{+/+}$ and $ASMase^{-/-}$ mice 24h after APAP administration (i.p. 300mg/Kg). Scale bar represents 50 µm.

Figure S2. Lack of additional mechanisms involved in the sensitization of ASMase null mice to APAP hepatotoxicity.

PMH from ASMase$^{-/-}$ mice and their WT littermates were treated with a 5 mM APAP dose *in vitro* at different incubation times and (a) phosphorylated JNK levels were analysed by western blot. Eight-week-old mice C57BL6 wild type (ASMase$^{+/+}$) and age-matched ASMase knockout mice (ASMase$^{-/-}$) were intraperitoneally injected with either APAP (300mg/Kg body weight) or equal volume of saline as vehicle control. (b) Acetylated lysines were analysed by western blot and (d) mRNA *Chop* levels were analysed by qPCR in total liver homogenates harvested 2h after APAP administration. (c) C-32 protein abundance and (e) nitrotyrosine protein adducts and were analysed by western blot in total liver homogenates harvested 6h after APAP injection. Data are expressed as mean ± SEM. n=3-6 animals per group. *p<0,05 vs vehicle-treated group.

Figure S3. Effect of ASMase deficiency or treatment with U18666A and 25-HC in lysosomal cholesterol homeostasis.

ASMase$^{+/+}$ and ASMase$^{-/-}$ PMH expressing Lamp-GFP were stained against free cholesterol with filipin (a) Representative confocal images of the staining. ASMase$^{+/+}$ showed enhanced cholesterol accumulation in lysosomes after U18666A treatment (1μg/ml) as seen in (b) the lysosomal colocalization analysis with filipin and (c) filipin intensity of confocal images. 25-HC (1μg/ml) treatment in ASMase$^{-/-}$ PMH was able to diminish lysosomal cholesterol content as observed by (d) filipin intensity analysis. Data are expressed as mean ± SEM of 3 independent experiments. *p<0,05 vs vehicle group; #p<0,05 vs U18666A treated group. Scale bar represents 20 μm.

Figure S4. Mitochondrial quality control in ASMase null mice after APAP exposure.

Mitochondrial DNA was isolated from ASMase$^{+/+}$ and ASMase$^{-/-}$ PMH treated with APAP 5 mM during 3h. (a) ND1 levels were analysed by PCR in this samples and normalized to LPL levels. Data was expressed relative to the vehicle-treated group of each genotype. (b) Reactive oxygen species release was detected following an incubation with DCF (10 μM) after 5 mM APAP overnight treatment in ASMase$^{+/+}$ and ASMase$^{-/-}$ PMH. Data are expressed as mean ± SEM; n=3-6 mice or 3 independent experiments. *p<0,05 vs vehicle-treated group; #p<0,05 vs APAP-treated ASMase$^{+/+}$ group.

Figure S5. **Expression levels of LAMP2, Rab7 and VAMP8 in ASMase null mice after APAP administration.**

Eight-week-old mice C57BL6 wild type (ASMase$^{+/+}$) and age-matched ASMase knockout mice (ASMase$^{-/-}$) were intraperitoneally injected with either APAP (300mg/Kg body weight) or equal volume of saline as vehicle control. (a) Lamp2, Rba7 and VAMP8 were analyzed by western blot in total liver homogenates harvested 2 hours after APAP injection and (b, c, d) the bands were quantified by densitometry using Image J software. Data are expressed as mean ± SEM; n=3 mice per group. *p<0,05 vs vehicle-treated group; #p<0,05 vs ASMase$^{+/+}$ group.

Figure S6. Protective effect of 25-HC in ASMase null mice and wild type mice against APAP hepatotoxicity.

Eight-week-old mice C57BL6 wild type (ASMase$^{+/+}$) and age-matched ASMase knockout mice (ASMase$^{-/-}$) were subcutaneously injected with a 30 mg/kg dose of 25-HC during 5 days. The last day, mice were intraperitoneally injected with either APAP (300mg/Kg body weight) or equal volume of saline as vehicle control. (a,c)

Representative images of H&E staining of ASMase$^{+/+}$liver sections harvested 6 hours after the APAP injection. (b,d) Serum ALT levels measured 6 hours after APAP injection. Data are expressed as mean ± SEM; n=5-9 mice per group. *p<0,05 vs vehicle-treated group; #p<0,05 vs APAP-treated group. Scale bar represents 100 μm.

Figure S7. Effect of amitriptyline and CBE in filipin staining and lysosomal staining.

ASMase$^{+/+}$ PMH expressing Lamp-GFP and mtKeima were overnight treated with Amitryptiline (10 μM) or CβE (50 μM) and free cholesterol was stained with filipin. Mitochondrial and lysosomal colocalization with free cholesterol (mtKeima-Lamp2-filipin) was analysed by confocal imaging. Images are representative of 3 independent experiments. Scale bar represents 20 μM.

Figure S8. Effect of amitriptyline and 25-HC in APAP hepatotoxicity and lysosomal colocalization with mitochondria.

ASMase$^{+/+}$ PMH expressing Lamp-GFP and mtKeima were overnight pre-treated with Amitryptiline (10 μM) or Amitryptiline + 25-HC (1μg/ml), and were incubated with a 5mM APAP dose during 3 hours. (a) Lysosomal colocalization with mitochondria was analysed by confocal imaging and (c) 5 images per treatment of 3 different experiments were analyzed with Image J to assess the percentage of lysosomal colocalization with mitochondria. (b) Cell viability was analyzed by Trypan Blue exclusion in ASMase$^{+/+}$ PMH pre-treated with Amitryptiline (10 μM) or Amitryptiline + 25-HC (1μg/ml) and exposed to a 15 mM APAP dose during 6 hours. Data are expressed as mean ± SEM of 3 independent experiments. *p<0,05 vs vehicle-treated group; #p<0,05 vs APAP-treated group and &p<0,05 vs APAP+Amitryptiline treated group. Scale bar represents 20 μm.

Figure S9. Prolonged treatment with amitryptiline or 25-HC does not affect APAP metabolism.

(a) Hepatic total GSH levels 1 hour after APAP treatment in ASMase$^{+/+}$ and ASMase$^{-/-}$ mice previously injected with amitriptyline (i.p. 5 mg/kg) or 25-HC (s.c. 30mg/kg) for 5 days. (b, c, d, e) phospho-JNK levels and CYP2E1 protein abundance in samples of liver tissues harvested 1h after APAP injection. Data are expressed as mean ± SEM (n=3) *p<0.05 vs ASMase$^{+/+}$ vehicle treated animals.

Figure S10 . Effect of CBE and 25-HC in in APAP hepatotoxicity and lysosomal colocalization with mitochondria.

ASMase$^{+/+}$ PMH expressing Lamp-GFP and mtKeima were overnight pre-treated with CβE (50 µM) or CβE + 25-HC (1µg/ml), and were incubated with a 5mM APAP dose during 3 hours. (a) Lysosomal colocalization with mitochondria was analysed by confocal imaging and (c) 5 images per treatment of 3 different experiments were analyzed with Image J to assess the percentage of lysosomal colocalization with mitochondria. (b) Cell viability was analyzed by Trypan Blue exclusion in ASMase$^{+/+}$ PMH pre-treated with CβE (50 µM) or CβE + 25-HC (1µg/ml) and exposed to a 15 mM APAP dose during 6 hours. Data are expressed as mean ± SEM of 3 independent experiments. *p<0,05 vs vehicle-treated group; #p<0,05 vs APAP-treated group and &p<0,05 vs APAP+Amitryptiline treated group. Scale bar represents 20 µm.

Figure S1.

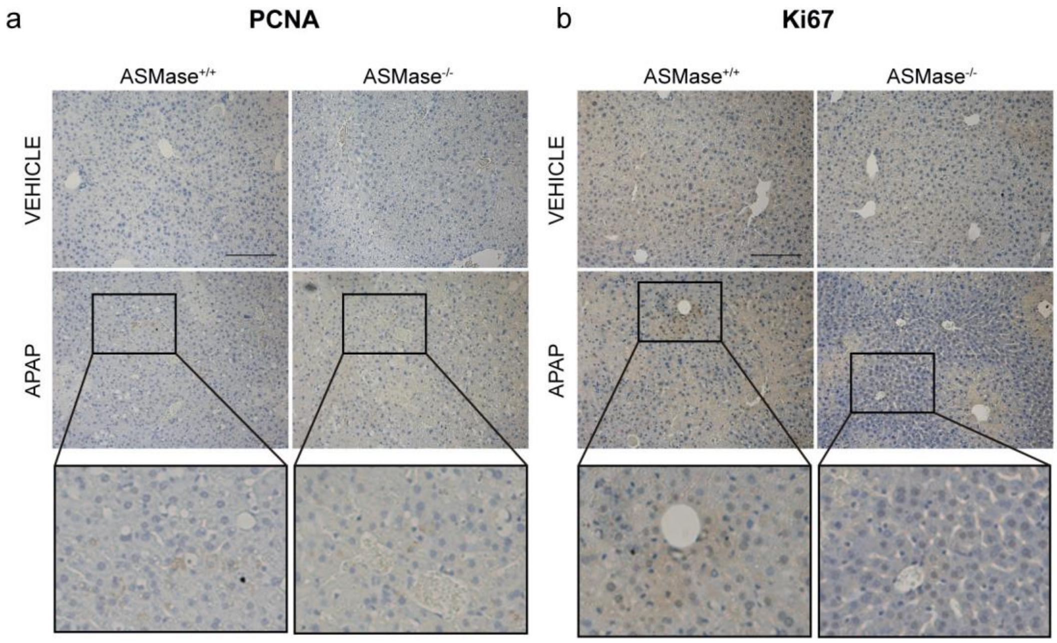
a
PCNA
ASMase+/+
ASMase-/-
VEHICLE
APAP
b
Ki67
ASMase+/+
ASMase-/-
VEHICLE
APAP

Figure S2.

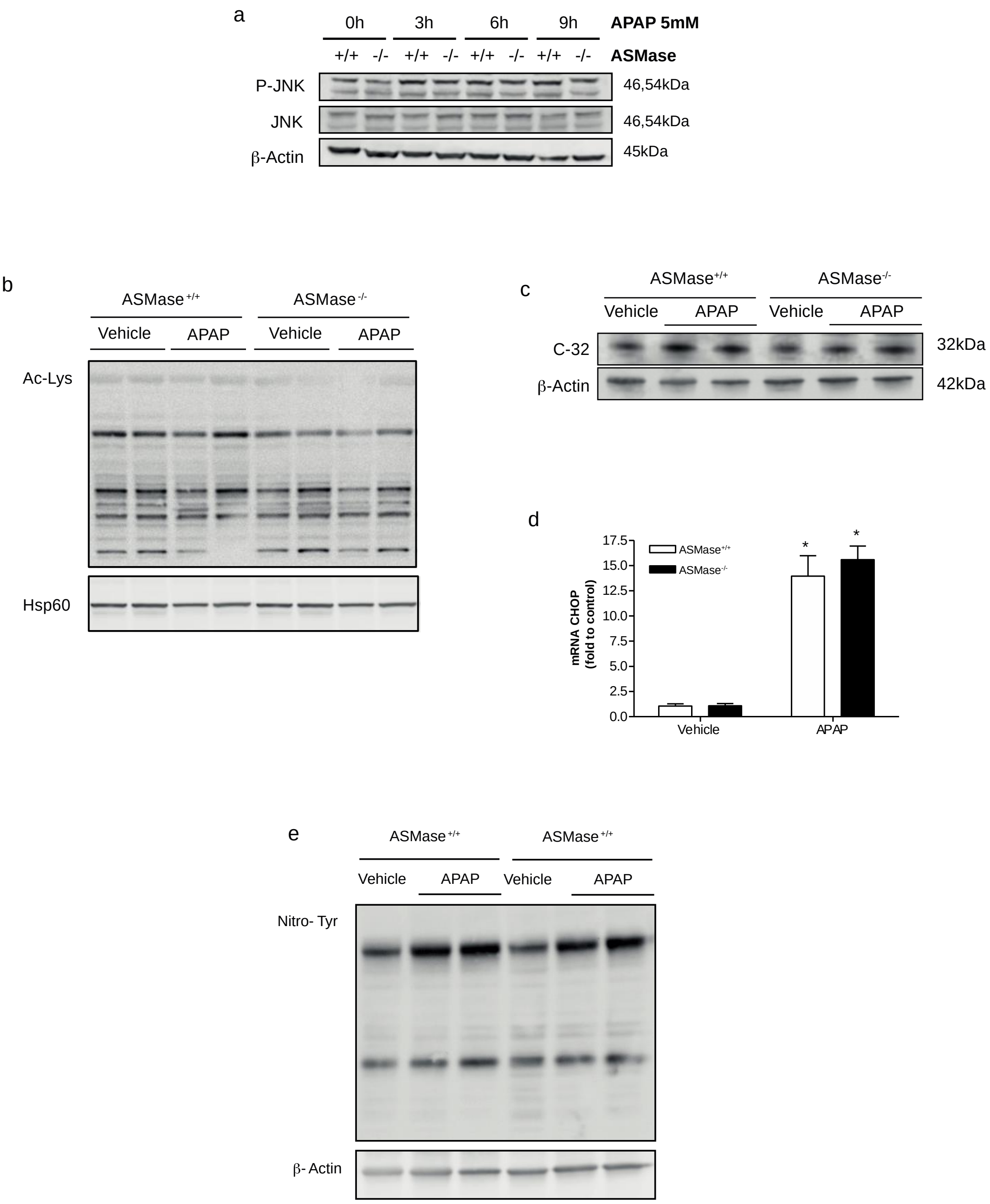
a
0h
3h
6h
9h
APAP 5mM
+/+ -/- +/+ -/- +/+ -/- +/+ -/-
ASMase
P-JNK
46,54kDa
JNK
46,54kDa
β-Actin
45kDa
b
ASMase +/+
ASMase -/-
Vehicle
APAP
Vehicle
APAP
Ac-Lys
Hsp60
c
ASMase+/+
ASMase-/-
Vehicle
APAP
Vehicle
APAP
C-32
32kDa
β-Actin
42kDa
d
ASMase+/+
ASMase-/-
mRNA CHOP
(fold to control)
17.5
15.0
12.5
10.0
7.5
5.0
2.5
0.0
Vehicle
APAP
e
ASMase +/+
ASMase +/+
Vehicle
APAP
Vehicle
APAP
Nitro- Tyr
β- Actin

Figure S3.

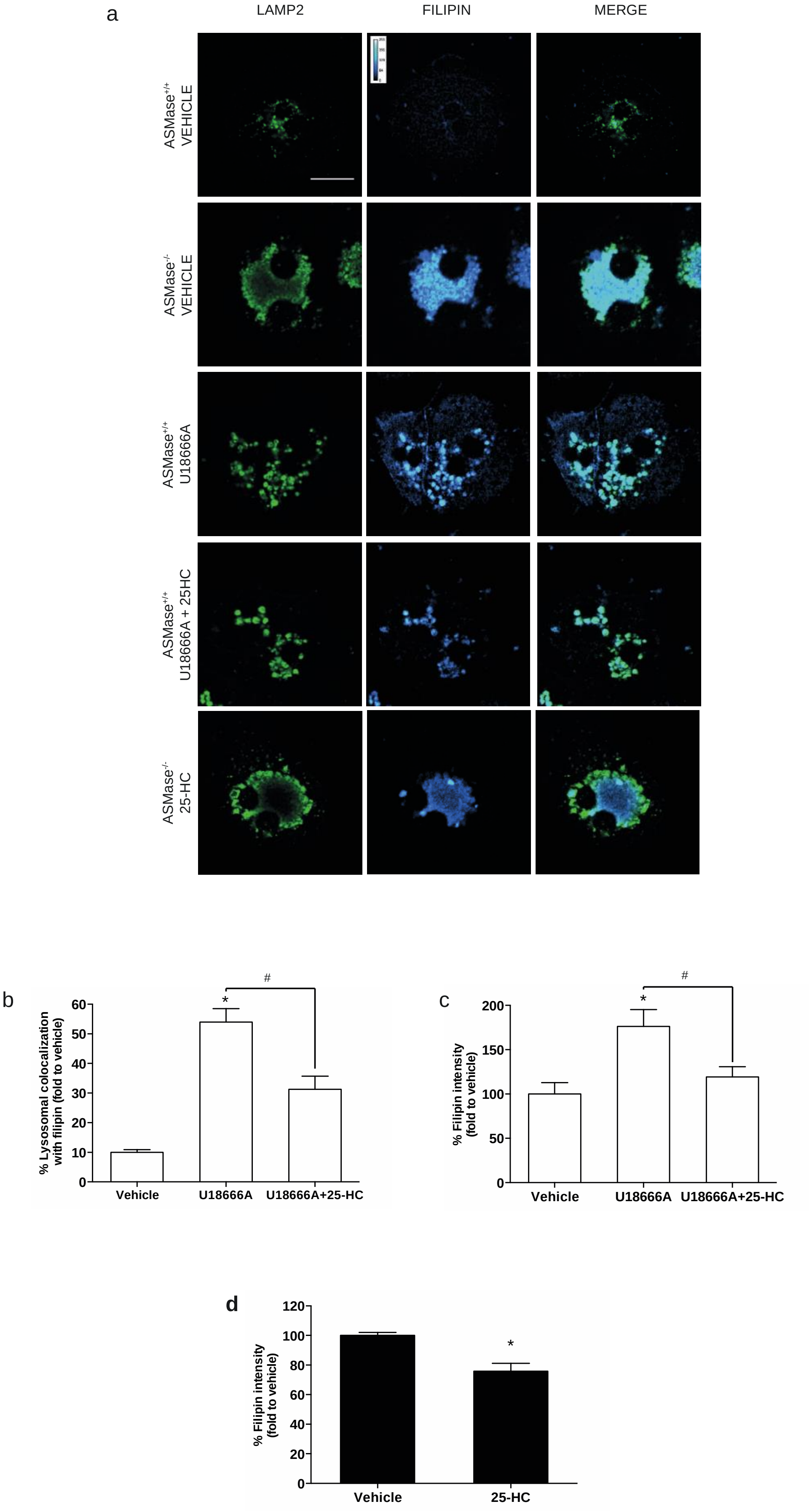
a
LAMP2
FILIPIN
MERGE
ASMase+/+ VEHICLE
ASMase-/- VEHICLE
ASMase+/+ U18666A
ASMase+/+ U18666A + 25HC
ASMase-/- 25-HC
b
% Lysosomal colocalization with filipin (fold to vehicle)
0
10
20
30
40
50
60
*
#
Vehicle
U18666A
U18666A+25-HC
c
% Filipin intensity (fold to vehicle)
0
50
100
150
200
*
#
Vehicle
U18666A
U18666A+25-HC
d
% Filipin intensity (fold to vehicle)
0
20
40
60
80
100
120
*
Vehicle
25-HC

Figure S4.

a

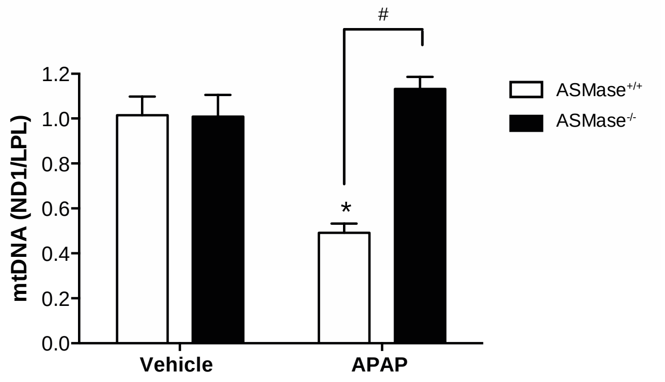
#
1.2
1.0
0.8
0.6
0.4
0.2
0.0
mtDNA (ND1/LPL)
*
Vehicle
APAP
ASMase+/+
ASMase-/-

b

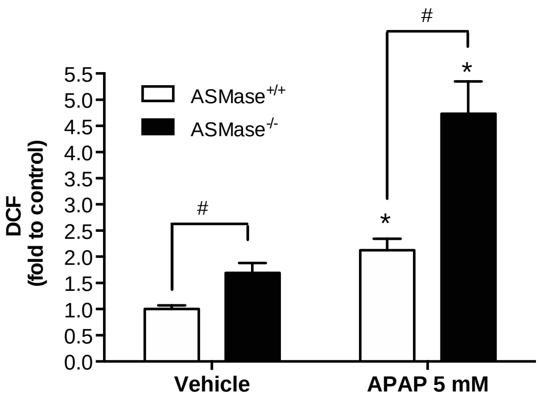
#
*
5.5
5.0
4.5
4.0
3.5
3.0
2.5
2.0
1.5
1.0
0.5
0.0
ASMase+/+
ASMase-/-
DCF
(fold to control)
#
*
Vehicle
APAP 5 mM

Figure S5.

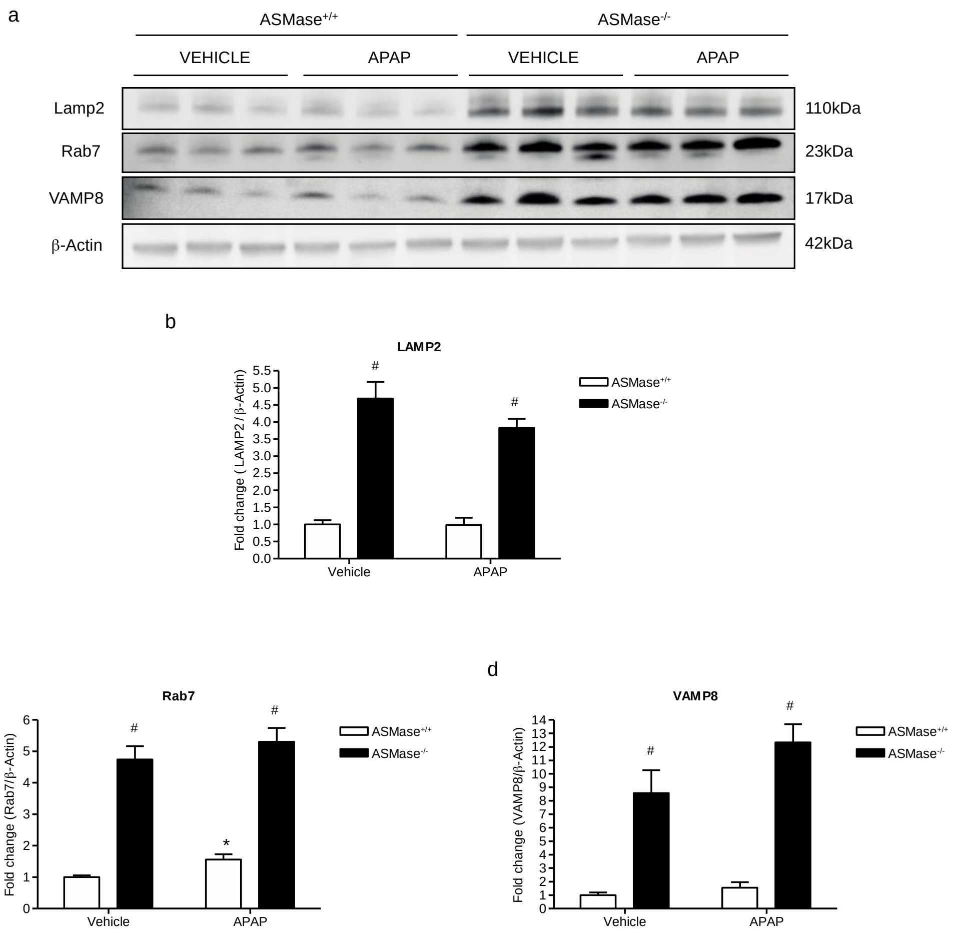
a
ASMase+/+
ASMase-/-
VEHICLE
APAP
VEHICLE
APAP
Lamp2
Rab7
VAMP8
β-Actin
110kDa
23kDa
17kDa
42kDa
b
LAMP2
Fold change (LAMP2 / β-Actin)
Vehicle
APAP
ASMase+/+
ASMase-/-
c
Rab7
Fold change (Rab7/β-Actin)
Vehicle
APAP
ASMase+/+
ASMase-/-
d
VAMP8
Fold change (VAMP8/β-Actin)
Vehicle
APAP
ASMase+/+
ASMase-/-

Figure S6.

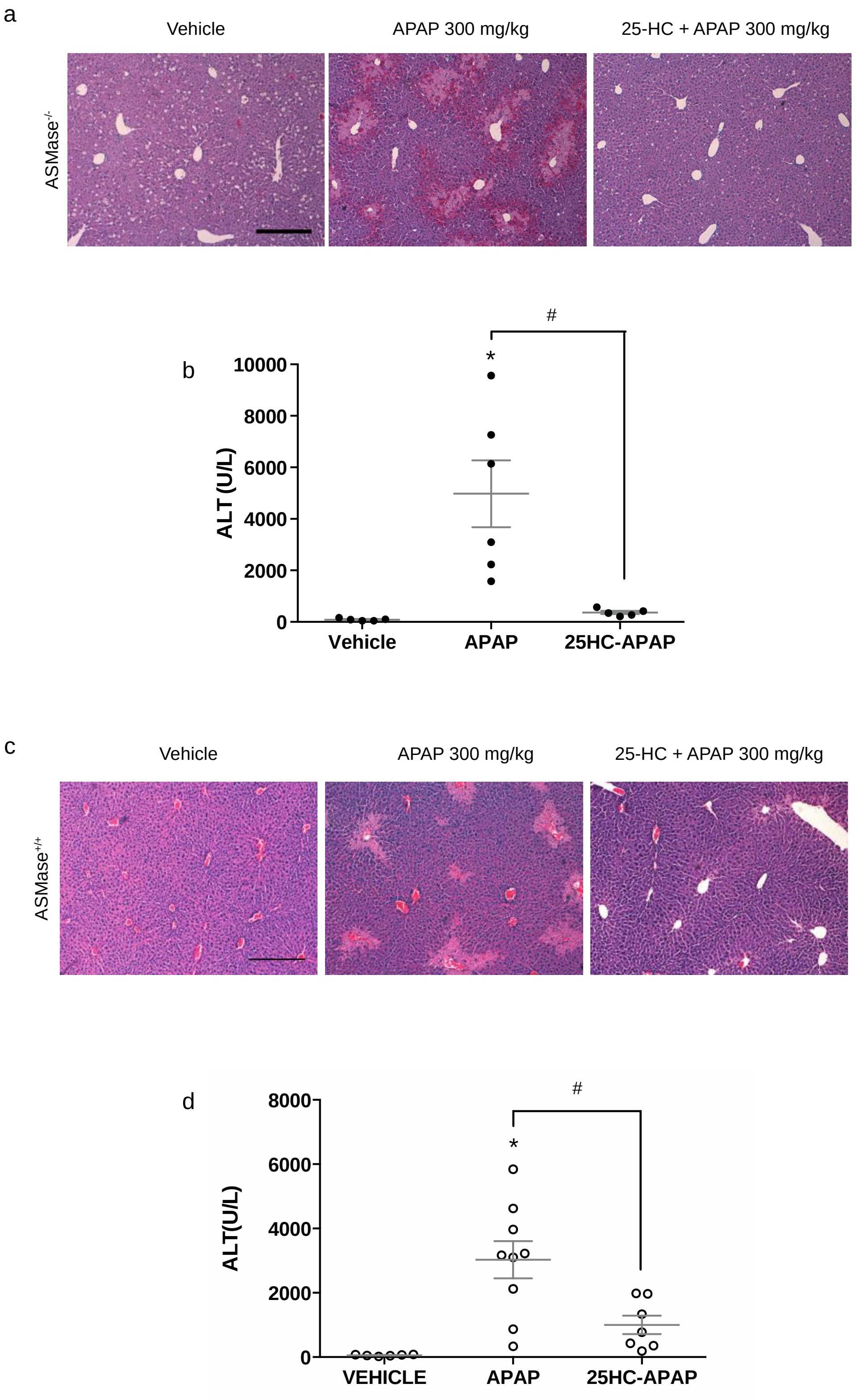
a
Vehicle
APAP 300 mg/kg
25-HC + APAP 300 mg/kg
ASMase-/-
b
ALT (U/L)
10000
8000
6000
4000
2000
0
Vehicle
APAP
25HC-APAP
*
#
c
Vehicle
APAP 300 mg/kg
25-HC + APAP 300 mg/kg
ASMase+/+
d
ALT(U/L)
8000
6000
4000
2000
0
VEHICLE
APAP
25HC-APAP
*
#

Figure S7.

a

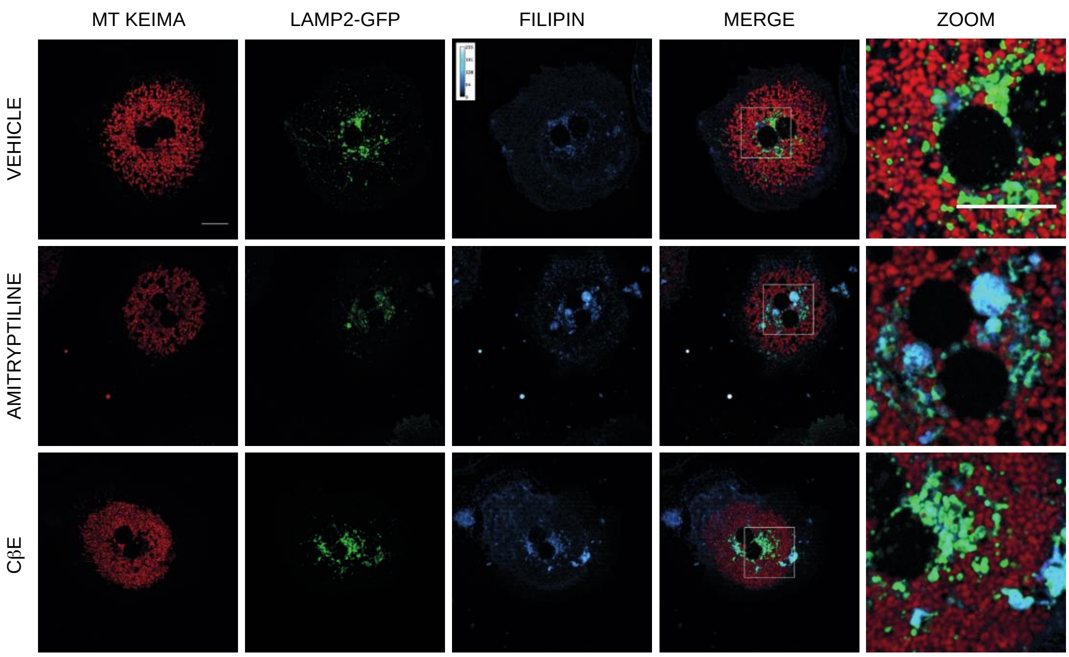
MT KEIMA
LAMP2-GFP
FILIPIN
MERGE
ZOOM
VEHICLE
AMITRYPTILINE
CβE

Figure S8.

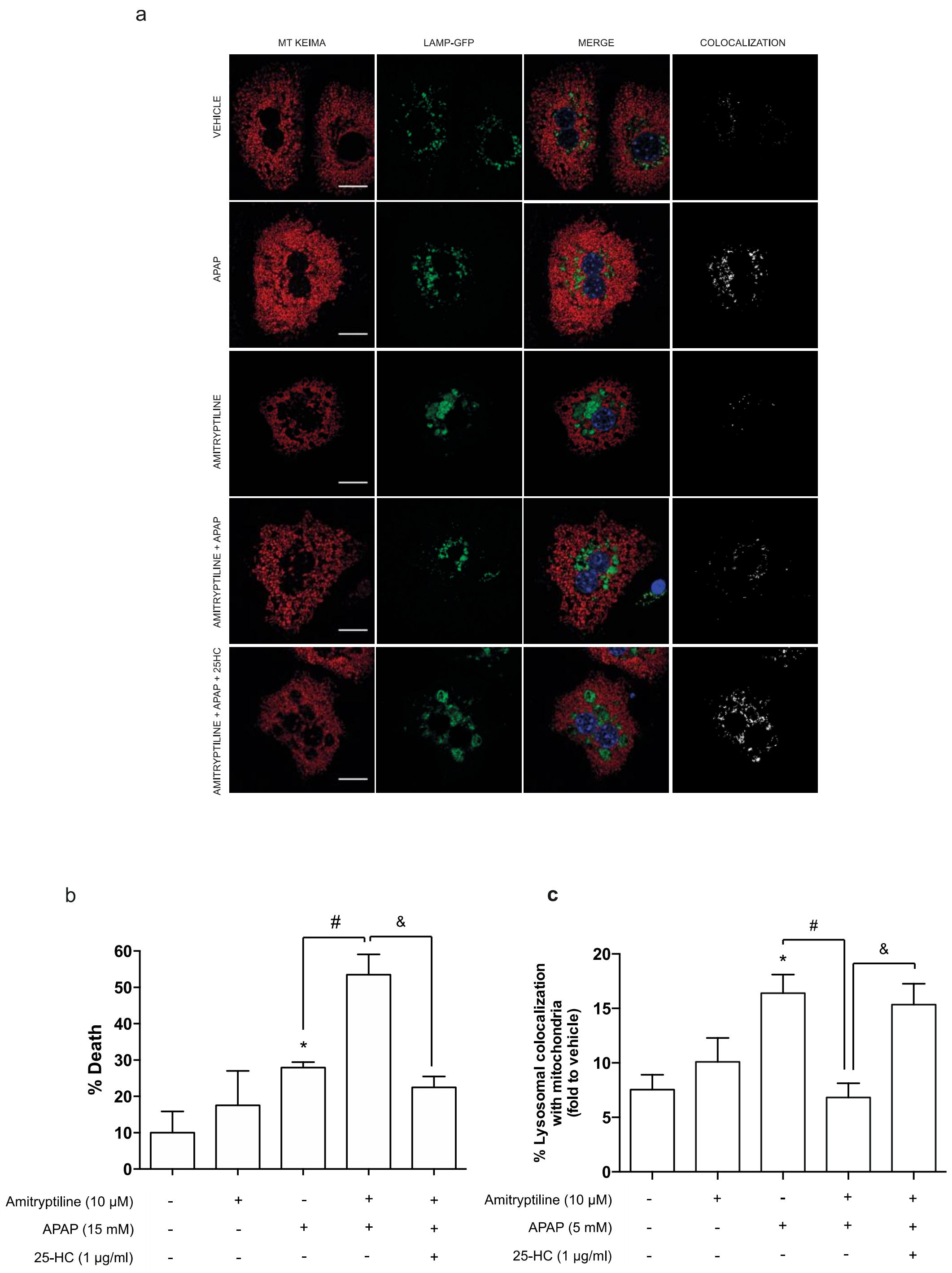
a
MT KEIMA
LAMP-GFP
MERGE
COLOCALIZATION
VEHICLE
APAP
AMITRYPTILINE
AMITRYPTILINE + APAP
AMITRYPTILINE + APAP + 25HC
b
% Death
#
&
*
Amitryptiline (10 μM)
APAP (15 mM)
25-HC (1 μg/ml)
c
% Lysosomal colocalization with mitochondria (fold to vehicle)
Amitryptiline (10 μM)
APAP (5 mM)
25-HC (1 μg/ml)

Figure S9.

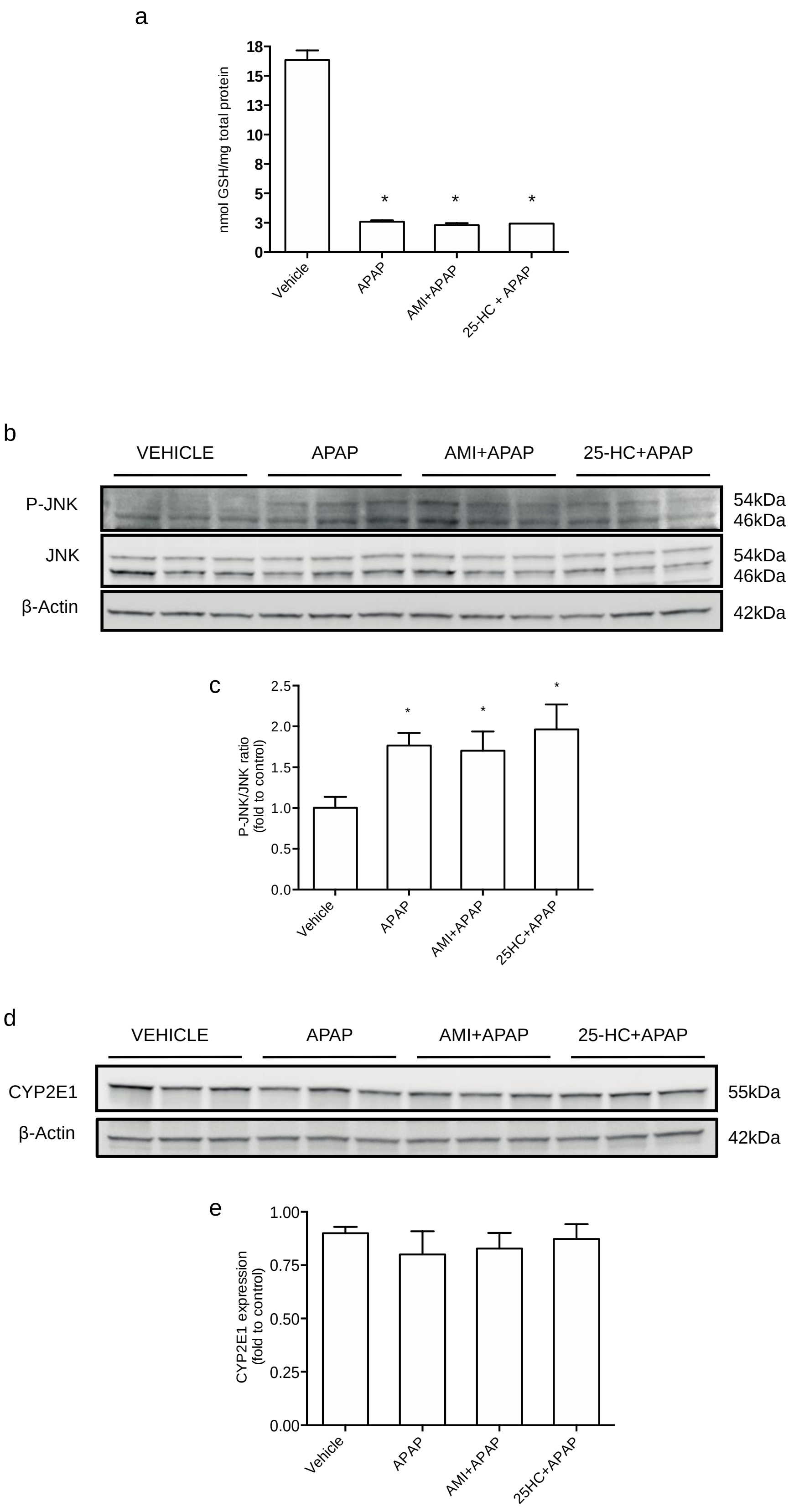
a
nmol GSH/mg total protein
18
15
13
10
8
5
3
0
*
*
*
Vehicle
APAP
AMI+APAP
25-HC + APAP
b
VEHICLE
APAP
AMI+APAP
25-HC+APAP
P-JNK
JNK
β-Actin
54kDa
46kDa
54kDa
46kDa
42kDa
c
P-JNK/JNK ratio
(fold to control)
2.5
2.0
1.5
1.0
0.5
0.0
*
*
*
Vehicle
APAP
AMI+APAP
25HC+APAP
d
VEHICLE
APAP
AMI+APAP
25-HC+APAP
CYP2E1
β-Actin
55kDa
42kDa
e
CYP2E1 expression
(fold to control)
1.00
0.75
0.50
0.25
0.00
Vehicle
APAP
AMI+APAP
25HC+APAP

Figure S10.

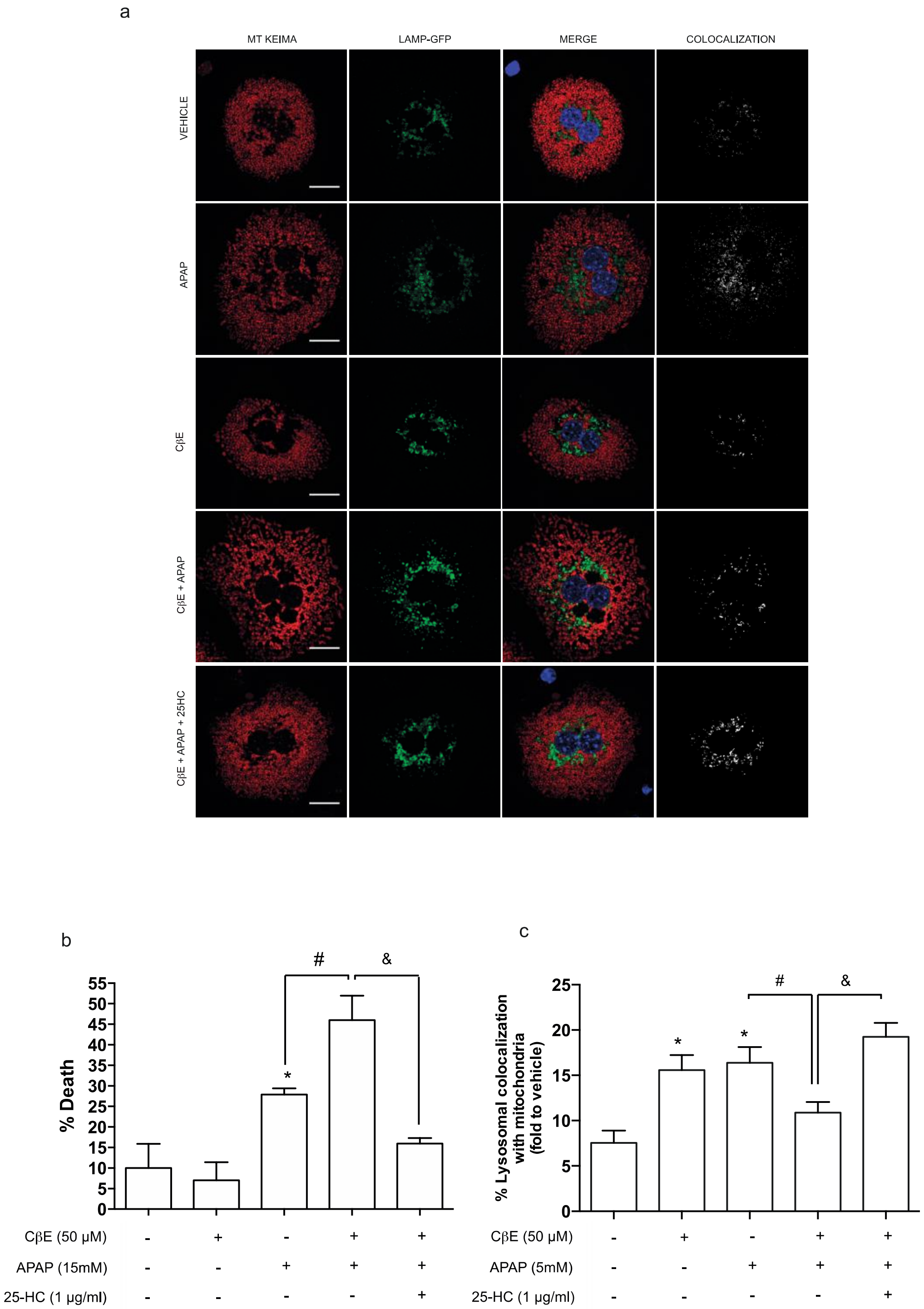
a
MT KEIMA
LAMP-GFP
MERGE
COLOCALIZATION
VEHICLE
APAP
CβE
CβE + APAP
CβE + APAP + 25HC
b
% Death
55
50
45
40
35
30
25
20
15
10
5
0
#
&
*
CβE (50 μM) - + - + +
APAP (15mM) - - + + +
25-HC (1 μg/ml) - - - - +
c
% Lysosomal colocalization with mitochondria (fold to vehicle)
25
20
15
10
5
0
#
&
*
*
CβE (50 μM) - + - + +
APAP (5mM) - - + + +
25-HC (1 μg/ml) - - - - +
